# Targeting Tumour Heterogeneity through sequential timing of anti-hallmark combination therapies -a hypothesis for implementation

**DOI:** 10.3389/fimmu.2026.1882175

**Published:** 2026-07-15

**Authors:** Kumara Swamy, Guruaj Arakeri, Ramaswamy Veena, Anirudh Vagata Srinivas, Amritanshu Ram, Agrahara Sreenivasa Kirthi Koushik, Naik Radheshyam, Rao Mohan Raghavendra, Basavalingaiah S. Ajaikumar

**Affiliations:** 1 Clinical Scientist Oncology, HealthCare Global Enterprises Ltd. (HCG), Bangalore, Karnataka, India; 2 Department of Head and Neck Oncology, Center for Academic Research, HCG Cancer Center, Bengaluru, Karnataka, India; 3 Histopathology Department, Triesta Sciences, HealthCare Global Enterprises Ltd., Bangalore, Karnataka, India; 4 Department of Radiation Oncology, HealthCare Global Enterprises Ltd. (HCG), Bangalore, Karnataka, India; 5 Yoga Department, HealthCare Global Enterprises Ltd., Bangalore, Karnataka, India; 6 Department of Medical Oncology, HealthCare Global Enterprises Ltd., Bangalore, Karnataka, India; 7 Research & Integrative Medicine, HealthCare Global Enterprises Ltd., Bangalore, Karnataka, India; 8 HealthCare Global Enterprises Ltd., Bangalore, Karnataka, India

**Keywords:** drug-tolerant persisters, epigenetic modifiers, epithelial-mesenchymal plasticity, hallmarks of cancer, immunotherapy, stereotactic body radiotherapy, tumor heterogeneity, vascular normalization

## Abstract

Since the introduction of the hallmarks of cancer framework over 25 years ago, treatment approaches have evolved into personalized medicine, offering benefits to select patient populations. However, three major components of heterotypic interactions in cancer—mutational evolution of cancer stem cells, epithelial-mesenchymal plasticity (EMP), and cancer-remodeled extracellular matrix (ECM)—remain critical barriers to therapy, particularly in patients who have failed treatment. EMP encompasses a spectrum of to-and-fro transitions between mesenchymal and epithelial states, yielding hybrid phenotypes of evolutionary heterogeneity. These are embedded in the vascular, metabolic, mutational, and immune-suppressive reprogramming of the tumor microenvironment (TME), induced and advanced by the hypoxia–reactive oxygen species (ROS)–hypoxia-inducible factor-1α (HIF-1α)–transforming growth factor-β (TGF-β) signaling axis. This review systematically examines the molecular mechanisms underlying EMP, tumor heterogeneity, and the hallmarks of cancer. It explores pharmacological strategies to target tumor burden, epigenetically revert transitional states, and restore immune-editing functions. Based on this analysis, we propose a phased anti-hallmark Combinations, Timing, and Sequencing (CTS) protocol. The methodology integrates vascular normalization, epigenetic modifiers, trimodal radiotherapy or stereotactic body radiotherapy (SBRT), chemotherapy (CT), and immunotherapy optimization, aiming to improve outcomes while minimizing toxicities. Also, mechanistically, by reverting mesenchymal phenotypes and normalizing the vasculature, the CTS protocol is designed to rescue the immune-suppressive tumor microenvironment—curtailing the recruitment of myeloid-derived suppressor cells (MDSCs) and regulatory T (Treg) cells. This restores cytotoxic T-cell homing, thereby converting immunologically “cold” tumors into “hot,” immunotherapy-responsive lesions.

## 1 Introduction

Tumor heterogeneity is fundamental to cancer cell survival and is the primary factor underlying therapy resistance. Major components include mutational heterogeneity in cancer stem cells, drawn from the intricate human genome, and the tumor-protective microenvironment (TME), characterized by a complex, heterogeneous extracellular matrix (ECM). These changes begin with reactive oxygen species (ROS) accumulation due to increased cellular stress, leading to stabilization of hypoxia-inducible factor-1α (HIF-1α), and are modulated by hypoxia, with one powering the other. Adding to this complexity, embedded within this heterogeneity lie epithelial–mesenchymal transition (EMT) and its reverse, mesenchymal–epithelial transition (MET), which transition to and from, resulting in epithelial–mesenchymal plasticity (EMP) in cancer (1–3). EMT, MET, and EMP are key regulators that underlie normal embryonic development and resurface in certain pathological conditions (4).

In cancer, polarized epithelial cells acquire a mesenchymal phenotype during EMT, characterized by loss of apical–basal polarity, reduced cell–cell adhesion, decreased expression of epithelial markers, and increased production of mesenchymal proteins. Tumor cells with enhanced mesenchymal traits acquire a migratory phenotype that promotes disease progression by breaching the basement membrane, infiltrating tissues, invading the vasculature, and ultimately facilitating metastasis. Beyond facilitating invasion, EMT confers stem cell-like properties and broad-spectrum resistance to chemotherapy (CT), radiotherapy (RT), and targeted agents (5).

After establishing a niche at the distant site in a favorable microenvironment, cancer cells with mesenchymal characteristics must undergo an evolutionary reversion to epithelial cells to proliferate at the site of seeding. This process occurs through MET, in which mesenchymal cells revert to a more epithelial-like state, gaining characteristics such as apical–basal polarity, enhanced adhesion, and reduced motility (6). In this process, EMT and MET are not binary transitions but represent a spectrum of intermediate phenotypes that profoundly influence cancer cell behavior. This EMP enables cancer cells to dynamically adapt to changing microenvironmental conditions. Additionally, therapy usually eliminates more susceptible epithelial cells, potentially exerting selective pressure on mesenchymal-type cells to evolve mutationally (7).

This review focuses on the dynamic interplay between EMT and MET in driving tumor invasion, stemness, and drug resistance within the hallmarks of cancer and in the context of tumor heterogeneity. The aim is to identify strategies to target tumor heterogeneity and adaptive EMT/MET plasticity in cancer cell populations, while simultaneously disrupting invasion-promoting EMT and MET-dependent proliferation dynamics (6, 8, 9). The article also explores the processes of epigenetic reversion, differentiation, and reprogramming (10). These phenomena require not only epigenetic reversions, as seen in MET, but also normalizing the TME to mimic embryonic microenvironments, without leaving an irreversible epigenetic damage scar in the ECM (11, 12). Essentially, the hypotheses proposed here are a synthesis of pre-priming antiangiogenic–EMT prevention combinations; priming therapy with RT–CT cancer/immune-suppressive cell depletion with vascular enhancement; and primary therapy with rapidly evolving immunotherapy–epigenetic modifier approaches. It bridges the established RT/SBRT-CT maximum reduction in tumor burden, optimizes the integration of immunotherapy, and anticipates the potential of epigenetic modifier adoption. RT and SBRT have advanced from focusing mainly on morphological traits to now incorporating the molecular EMT-MET-mutational evolution of cancer, immune modulation, and memory cell pool expansion strategies to prevent recurrences. The other proposed strategy is differentiation therapy, i.e., the process of pushing malignant, plastic cells out of cycling, resistant states into terminal, post-mitotic, benign lineages (such as adipocytes or osteoblasts)—an intervention detailed in later sections (13, 14). Reverting EMP phenotypes is not merely a cancer-cell-intrinsic maneuver. It also attenuates mesenchymal-driven immunosuppressive signaling, which is expected to curtail the EMT-associated recruitment of myeloid-derived suppressor cells (MDSCs) and regulatory T (Treg) cells. This restores cytotoxic T-cell homing into the tumor, thereby helping to rescue the immune-suppressive TME that the CTS protocol ultimately seeks to reset (15, 16).

## 2 Cancer initiation, progression, transition, stemness, and ecological evolution

### 2.1 Primary drivers of tumor heterogeneity and EMP

The cascading molecular cellular stress process is the reason for the initiation and progression of cancer; understanding of which becomes essential in targeting and epigenetic reversion of cancer.

#### 2.1.1 ROS–hypoxia stress

**Figure 1:**
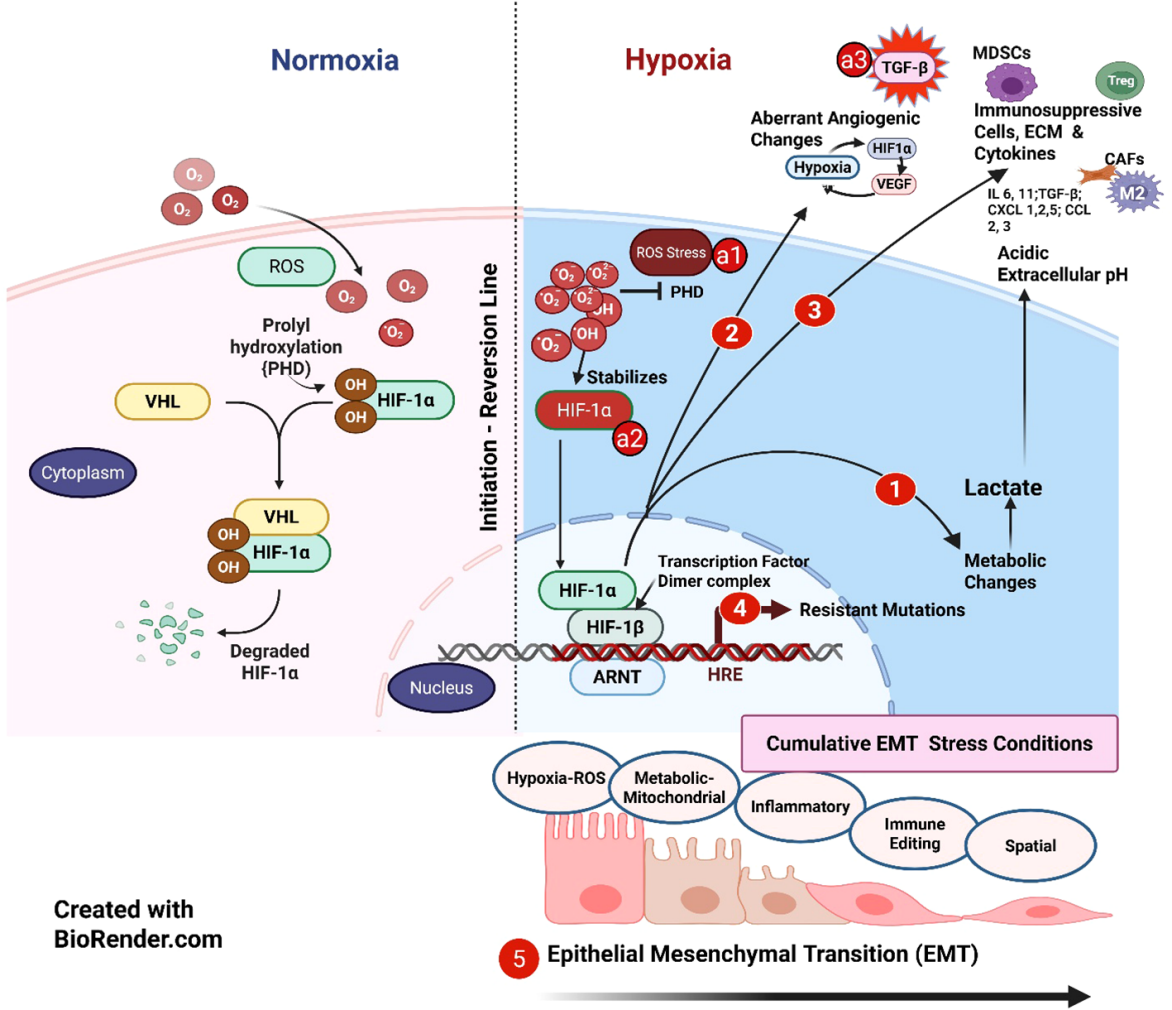
Evolution of heterogeneity. In normoxic conditions, prolyl hydroxylases (PHDs) hydroxylate HIF-1α, thereby relieving ROS-induced oxidative stress. Von Hippel–Lindau (VHL) protein marks for degradation. In a hypoxic environment, PHDs are inhibited, HIF-1α is stabilized, and HIF-1α accumulates in the cytosol. Stabilized HIF-1α enters the nucleus, forming a dimer with HIF-1β, influencing numerous homeostatic genes and leading to cascading effects in cancer progression: (1) Warburg effect; (2) VEGF-driven aberrant neoangiogenesis; (3) genomic mutations driving diverse clones; (4) accumulation of immunosuppressive cells, CAFs, and cytokines in the TME; (5) EMT and pathways of stemness, metastases, and resistance. a1–a2–a3: ROS–HIF-1α–TGF-β foundational axis. IL, interleukin; CXCL, chemokine C-X-C motif ligand; CCL, C-C motif ligand; MDSCs, myeloid-derived suppressor cells; M2, tumor-associated macrophage 2; Treg, regulatory T cells. Created in BioRender. Swamy K (2026) https://BioRender.com/mvvzkpi.

Oxidative stress in the cell leads to excessive accumulation of ROS, which triggers HIF-1α stabilization (**Figure 1**). Stabilized HIF-1α in this pseudohypoxic state affects mitochondrial and glucose metabolism (17, 18). **Figure 1** maps the subsequent events (19). The onset of hypoxia, downstream effects of aberrant vasculature (including co-option/vascular mimicry), metabolic reprogramming, the acquisition of cancer stem cell traits, the induction of cancer-associated fibroblasts (CAFs), and immune dysregulation lead to cancer heterogeneity with core-resistant phenotypes. Cancer cells maintain a state of “partial EMT,” initially characterized by retention of some epithelial characteristics while acquiring increased migratory capabilities, eventually leading to EMT-driven infiltration (15, 20). Hypoxia affects pericyte coverage, promoting the invasiveness of EMT cancer cells (21, 22). With the onset of EMT/EMP/metastasis, secondary mutational resistance sets in (23, 24). Tumor hypoxia also induces angiogenic dormancy, autophagy, and lymphangiogenesis, with increasing therapy resistance (19). Importantly, hypoxia does not merely induce EMT; the lactate-driven acidification of this niche fundamentally alters the local metabolic microenvironment and directly paralyzes infiltrating cytotoxic T lymphocytes (CTLs). Lowering the extracellular pH to the values that characterize tumor masses (pH 6–6.5) is sufficient to establish an anergic state in CD8+ tumor-infiltrating lymphocytes, with impaired cytolytic activity, reduced cytokine secretion, and diminished T-cell receptor signaling, all of which are reversible upon pH restoration (25). This metabolic immunosuppression therefore promotes the immune evasion described in Section 2.1.3.

#### 2.1.2 Metabolic and mitochondrial stress

The heterodimer of HIF-1α and HIF-1β (**Figure 1**) promotes the expression of a series of target genes, including glucose transporters (GLUTs) and the glycolytic enzyme lactate dehydrogenase A (LDHA). LDHA accelerates the conversion of pyruvate to lactate, the Warburg effect, facilitating the proliferation of cancer cells (26). Lactate, a byproduct of glycolysis, diffuses into the extracellular TME, leading to a progressively more acidic pH. This increasing extracellular acidosis promotes immunosuppression, rendering several anticancer drugs ineffective. The acidic pH also fosters genomic mutations and further fuels tumor growth and the development of resistant phenotypic subclones (**Figure 1**) (27, 28). As the metabolic stress increases, proliferating cancer cells run out of nutrients, prompting them to undergo TGF-β-induced EMT, thereby moving away from nutrient-depleted locations (29). Beyond fostering immunosuppression, this low extracellular pH directly blunts the efficacy of weakly basic chemotherapeutic agents through a process known as “ion trapping”. Protonation of the drug in the acidic interstitium reduces its cellular uptake, providing a direct physicochemical pharmacokinetic mechanism for therapy resistance (30).

#### 2.1.3 Immune editing stress and avoidance of immune destruction

Immunoediting theory describes a three-step process of immunosurveillance, equilibrium, and immune escape, involving evasion of T-cell homing and immune destruction (31, 32). Cytotoxic CD8+ T cells initially eliminate proliferating cancer cells during immunosurveillance and later reach equilibrium as stress increases. If the carcinogenesis process continues, within this immune editing loop, cytotoxic T cells are reprogrammed into immunosuppressive cells that allow cancer cells to evade immune destruction (immune escape), leading to proliferation, increased stemness, and therapy resistance (16, 33).

#### 2.1.4 Inflammatory stress and the role of TGF-β

**Figure 2:**
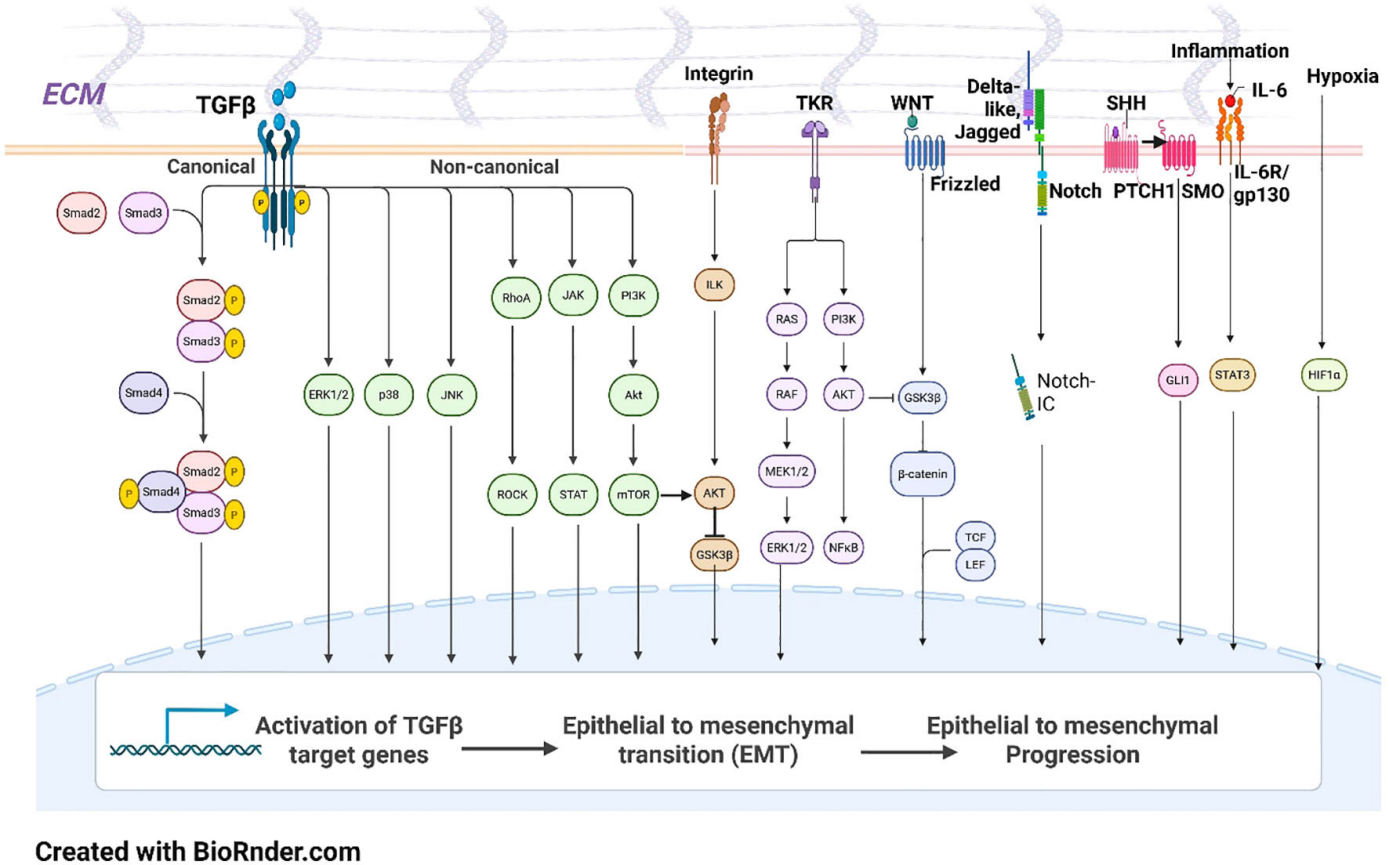
Signaling pathways of TGF-β activation, EMT, and progression. ECM, extracellular matrix; TGF-β, transforming growth factor-beta; TKR, tyrosine kinase receptors; WNT, Wingless/Integrated family of genes; SHH, Sonic Hedgehog; PTCH1, Patched-1 protein; SMO, Smoothened, Frizzled family receptor; IL, interleukin; GP, glycoprotein. Downstream = transcription factors (see text). Created in BioRender. Swamy K (2026) https://BioRender.com/19zifb7.

Inflammatory microenvironment (iTME) stress drives carcinogenesis, immune escape, and progression, exacerbated by oxidative stress (ROS). The primary factor identified is the multifunctional cytokine TGF-β, which initially plays a role in tumor suppression but later becomes fundamental to tumor progression, EMT, and stemness. The less understood “TGF-β switch” activates the Snail and Smad pathways, leading to EMT (**Figure 2**). In addition, over time, TGF-β acquires multiple tumor-promoting effects, increasing ECM fibrosis and rigidity and preventing immunological cross-talk. Subsequently, cells such as CAFs, tumor-associated macrophages (TAMs), T helper 17 (Th17) cells, and regulatory T (Treg) cells, as well as proinflammatory cytokines such as interleukin (IL)-6 and IL-8, sustain EMT (34). Janus kinase/signal transducer and activator of transcription (JAK/STAT) and nuclear factor-κB (NF-κB) pathways have also been implicated in maintaining mesenchymal traits and supporting EMT-driven tumor progression (15, 20, 35). This dual, paradoxical behavior of TGF-β merits emphasis. Early in tumorigenesis, it acts as a potent tumor suppressor that restrains cell-cycle progression and can even enforce a lethal EMT in premalignant cells; only later does it become an oncogenic, pro-EMT driver. The molecular “switch” governing this conversion is typically led by the mutational inactivation of parallel tumor-suppressor circuits (for example, p53) or by hyperactivation of oncogenic RAS signaling, which uncouples TGF-β from its cytostatic and pro-apoptotic outputs (36, 37).

#### 2.1.5 Spatial and compression stress

Proliferation of cancer cells within the given space leads to compressive pressure on each other. Stagnant blood flow, decreased lymphatic drainage, and CAF proliferation, which increase interstitial pressure, create physical barriers (7). ECM and the cell–stromal communication system change in response to stiffness and altered shear forces. This is an example of mechanotransduction, in which mechanical cues are sensed and transmitted, and the resulting signals are converted into biochemical signals that promote tumor invasion and metastasis through EMT (38). The reduction in spatial stress due to treatment intervention and the clearance of dead cancer cells significantly disrupt mechanical barriers and improve ECM softness (39).

**Figure 1** depicts the various stress mechanisms that lead to the initiation and multistage, multipathway progression of cancer, including EMT.

#### 2.1.6 Evolutionary mutational stress and the therapeutic “predator–prey” game

Secondary resistance can develop during treatment itself, and the predator–prey dynamic could favor the “prey” (cancer), armed with access to the vast information in the human genome (40). Within the tumor ecosystem, cancer cells exhibit ecological, spatial, and temporal Darwinian dynamics of natural selection, leading to the emergence of diverse cell subpopulations before and after anticancer therapy. The evolutionary changes involve heritable genomic mutations such as chromosomal rearrangements, gene duplication, and aneuploidy (40).

This “evolutionary rescue” can occur even in a small, rapidly declining population of cancer cells, after the lysis and clearance of the bulk of sensitive cells; secondly, due to the respite these cells receive to recover; and thirdly, due to exposure to sublethal drug concentrations that facilitate acclimatization, or following adaptive drug efflux by cancer cells. At the molecular level, membrane extrusion pumps may amplify suboptimal drug concentrations, leading to multidrug resistance (MDR). A classic example of success in addressing these issues is the consistent sequencing of multiple drugs in lymphoma to overcome MDR. However, these eco-evolutionary aspects are less thoroughly explored in the literature (40, 41).

### 2.2 Molecular drivers of EMT-MET heterogeneity

#### 2.2.1 Key signaling pathways

Among the most significant is TGF-β signaling, a well-established inducer of EMT across various cancer types. TGF-β activates Smad proteins, which enter the nucleus and regulate the transcription of EMT-related genes, thereby leading to cytoskeletal reorganization and increased cellular motility (42). Other targetable influential pathways are depicted in **Figure 2** (43, 44).

#### 2.2.2 Transcription factors

EMT transcription factors (EMT-TFs) are instrumental in orchestrating EMT. The Snail family (SNAI1 and SNAI2), zinc finger E-box binding homeobox (ZEB) family (ZEB1 and ZEB2), and twist family bHLH transcription factor (TWIST1) are prominent transcriptional regulators that inhibit the expression of E-cadherin, upregulate N-cadherin, and promote mesenchymal gene expression (45).

#### 2.2.3 Role of microRNA

MicroRNA-200c (miR-200c) exerts context-dependent actions that enhance apoptosis, inhibit tumor growth, reduce cellular inflammation, and suppress pyroptosis. In other situations, it can influence EMT promotion, modulate the TME via M2-phenotypic macrophage polarization, the density of tumor-infiltrating lymphocytes (TILs), programmed death-ligand 1 (PD-L1) expression, cytotoxic T lymphocyte (CTL) exhaustion, and the cancer cell exosomal load. MiR-200c can thus act as both a biomarker and an EMT progression/reversion factor (9, 46, 47). Central to this behavior is the highly conserved, double-negative feedback loop between the miR-200 family and the ZEB transcription factors, in which ZEB1/ZEB2 transcriptionally repress miR-200 while the miR-200 members reciprocally suppress ZEB translation. This ZEB/miR-200 loop functions as the fundamental biochemical switch that determines whether a cell rests in either a predominantly epithelial state, a mesenchymal state, or a highly plastic multiple-intermediate (hybrid/metastable) transitional state between these two endpoints. This underpins the entire EMP spectrum discussed throughout this review (9, 48).

### 2.3 Mechanisms and impact of MET

Although the exact molecular mechanism remains to be elucidated, the EMT-MET transition is accompanied by the reestablishment of cell–cell junctions and the regain of epithelial markers, thereby providing an environment essential for the resumption of proliferation in new tissues (49, 50). Agents that induce MET can restore epithelial characteristics in cancer cells at the primary site, potentially reduce metastatic potential, and enhance treatment sensitivity (51, 52). Repressed E-cadherin expression, a hallmark of EMT, is restored upon re-establishment of cell–cell junctions and chromatin accessibility, thereby impairing the migratory capabilities of cancer cells and promoting sensitivity (51). Reversible surface E-cadherin and cellular gene expression dynamics are closely related to TGF-β levels during *in vitro* studies (52).

**Figure 3:**
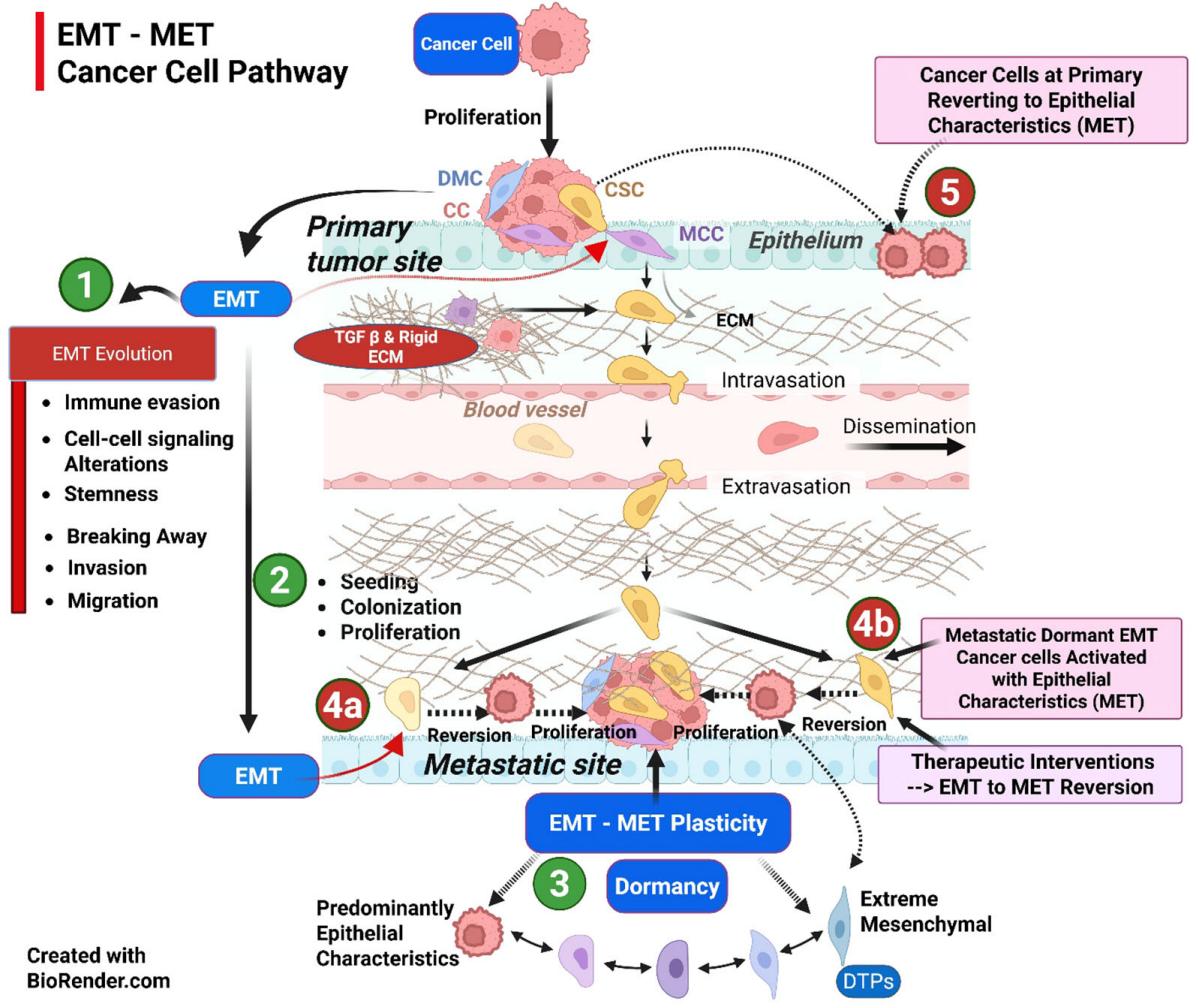
Mesenchymal–epithelial transition (MET) cancer cell pathways of initiation, invasion, dissemination (1); seeding/colonization (2); development of EMP (3); reversion to MET of seeded/dormant EMT/EMP cells (4a,b) or at primary site (5). DMC, DNA methylation changes; CSC, cancer stem cell; CC, cell competition; MCC, mutated colorectal cancer oncogene; TGF-β, transforming growth factor-beta; DTPs, drug-tolerant persister cells. Created in BioRender. Swamy K (2026) https://BioRender.com/0288ysz.

The dynamic interplay between EMT and MET enables cancer cells to adapt to various microenvironments throughout cancer progression (**Figure 3**). Circulating tumor cells have been found to display both epithelial and mesenchymal markers, indicating a hybrid population (53, 54). Thus, EMT, when the milieu is stressful (infiltration and metastasis), and MET, when the milieu is fertile (proliferation), are the foundational survival mechanisms by which cancer cells adapt, progress, evolve, and metastasize. *In vitro* studies show that the addition of bone morphogenetic protein 7 (BMP7), the reduction of TGF-β, and reduced hypoxia encourage MET (55). EMT–MET connections are spatially separated during the metastatic seeding process and undergo multiple pathophysiological and epigenetic changes (56). Therefore, “reversion” may be a more appropriate term than “reversal,” since cancer cells may not return to their true original state of all epithelial characteristics via MET (10). Accordingly, the term “reversion” (rather than “reversal”) is used consistently throughout the remainder of this article, including in the contexts of EMT/MET and epigenetic reversion. During this interim period, MET reversion at the metastatic site can also exhibit higher stemness, being more resistant than that of primary epithelial cells, yet sharing predominantly similar characteristics (54). As recently observed, senescent cells, considered irreversibly phenotypic, can be reactivated to secrete promigratory cytokines or re-enter the cell cycle under a deteriorating TME (41).

### 2.4 EMT-MET spectrum: epithelial-mesenchymal plasticity characterization

**Figure 4:**
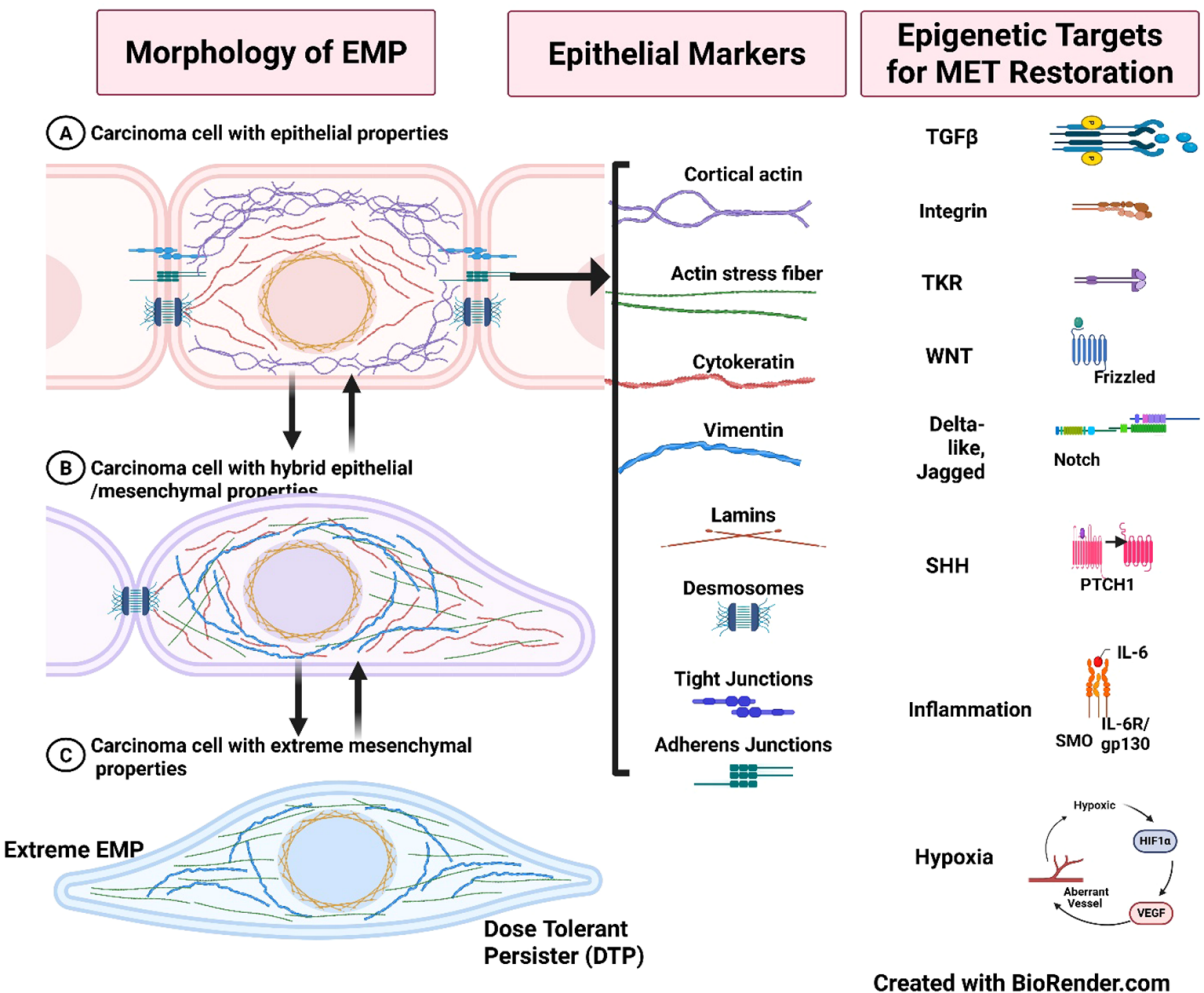
EMP Morphology and Epigenetic Targets for Restoring Epithelial IdentityThree-state model of epithelial-mesenchymal plasticity in carcinoma progression **(A–C)** with corresponding epithelial biomarkers and therapeutic epigenetic targets for reversing mesenchymal phenotypes and eliminating therapy-resistant persister cells. (Created with BioRender.com; Swamy K, 2026).

The cancer mass exhibits a spectrum of cells spanning the EMT-MET profile, classified as EMP (**Figure 4**). EMP hybrid features of cancer make it a diverse, shifting target as part of tumor aggressiveness and therapeutic resistance (57, 58). Although EMP accounts for only a fraction of the total number of cancer cells, its complexity is rife with controversy due to a lack of irrefutable evidence. Several terms exist in the literature: partial EMP (not committed to, thus exploiting both polar states), incomplete EMP (just short of full transition), resulting in a hybrid state (coexpression of both); transient de-differentiation (EMT)–re-differentiation (MET) switch states; or metastable (relatively fixed somewhere in the EMP axis spectrum). Characterization of EMT and MET thus provides invaluable insights into cancer biology (59, 60).

Additionally, the interconnected roles of EMT-related transcription factors and cancer stem cell (CSC) markers (e.g., CD44, aldehyde dehydrogenase (ALDH)) hint at a complex regulatory network that underscores not only the plasticity but also the adaptability of carcinoma cells (49). Epithelial markers such as E-cadherin, cytokeratins, and occludins are often diminished in advanced stages of cancer. In contrast, mesenchymal markers, including vimentin, N-cadherin, and fibronectin, gain prominence, indicating a different profile for evaluation across cancer stages (46, 61). Immunohistochemical profiling of these markers can provide insights into tumor EMT status, assisting prognostic evaluations and therapeutic decisions (62).

Other features in cancer cell evolution include dedifferentiation and redifferentiation, as well as changes in cancer stem cell resetting. The term transdifferentiation refers to another process of MET, in which cells acquire favorable features of different lineages, such as breast cancer cells transforming into adipocytes upon targeted induction (13).

## 3 Clinical implications of EMP

The presence of EMP post-therapy, compared with pre-therapy, is a poor prognostic factor (59). Evaluating EMP and stemness before neoadjuvant therapy and in postoperative surgical specimens would indicate an effective or improved protocol.

Caution is warranted when encouraging EMT-to-MET transition, as it may inadvertently induce MET-driven proliferation of cancer cells, including cancer stem cells. Therefore, MET induction should simultaneously target proliferating MET cells and sensitize cancer EMT/stem cells, underscoring the importance of targeting the epigenetic regulators of the interconversion between EMT and MET, along with the cellular plasticity between them as a continuum. An alternative approach is to induce extreme EMT reversion to terminal differentiation/apoptosis, which can be achieved with epigenetic modifiers (6). That exploiting EMP for terminal differentiation is a viable strategy rather than a purely theoretical construct is demonstrated by groundbreaking preclinical work in which invasive breast cancer cells actively undergoing EMT were transdifferentiated into post-mitotic benign adipocytes using a combination of the antidiabetic PPARγ agonist rosiglitazone and a MEK inhibitor (13).

The advantage of the MET reversion strategy upfront is that it can improve sensitivity before timed standard therapy—CT/RT/stereotactic body radiotherapy (SBRT)—and prevent EMT induction by the standard therapies (4, 63), thus targeting the double-bind situation of progression by MET versus metastasis with EMT.

The ECM is as critical as cancer cell features in EMP/stemness and needs to be normalized to improve therapeutic response. The stromal component plays a significant role in MET in addition to intrinsic factors (64).

## 4 Tumor heterogeneity, CSCS, and EMP—clonal cooperation

**Figure 5:**
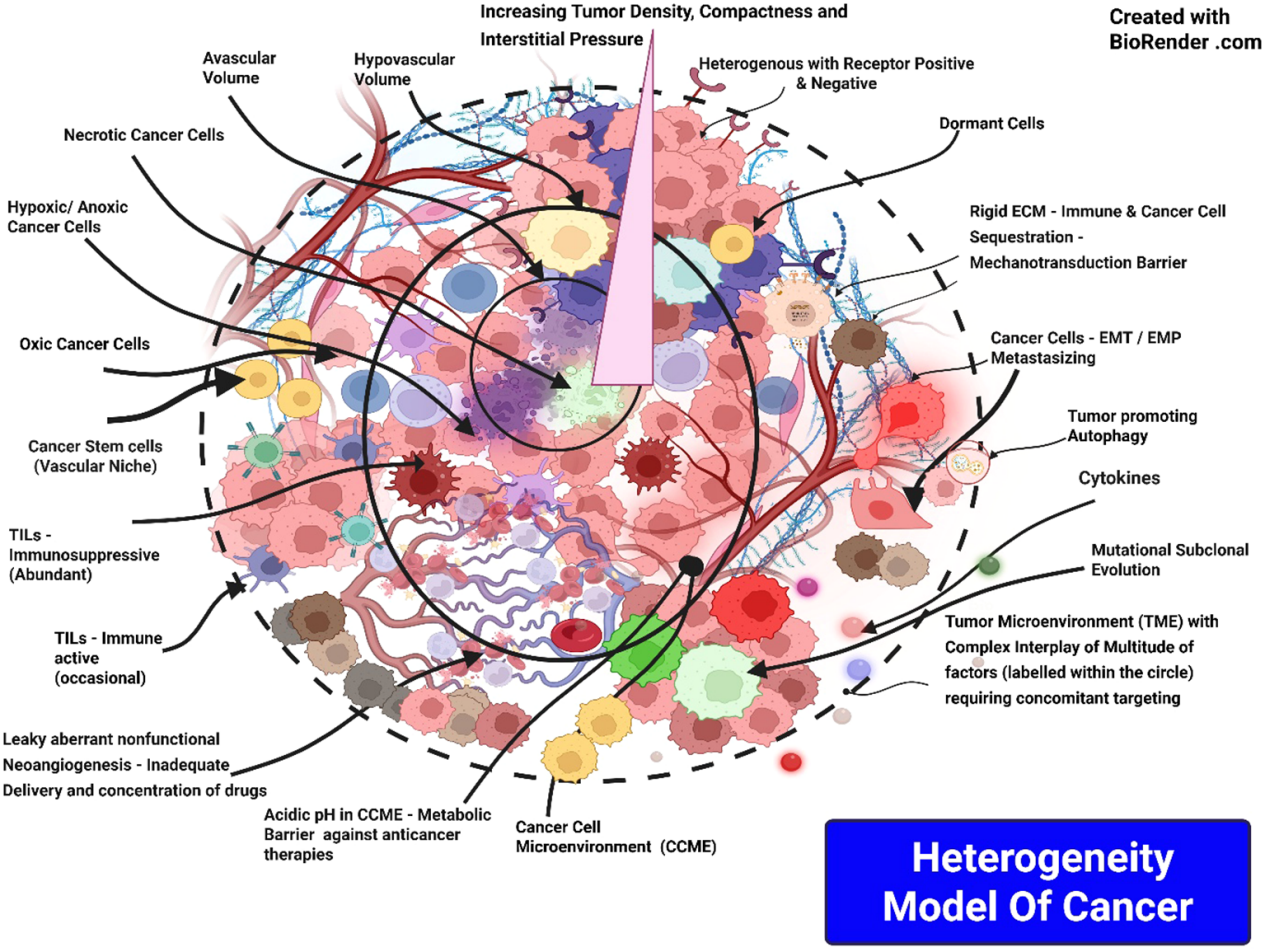
Diverse cancer heterogeneity compartments. TILs, tumor-infiltrating lymphocytes; ECM, extracellular matrix; EMT, epithelial-mesenchymal transition. Created in BioRender. Swamy K (2026) https://BioRender.com/n1q5ulq.

**Figure 6:**
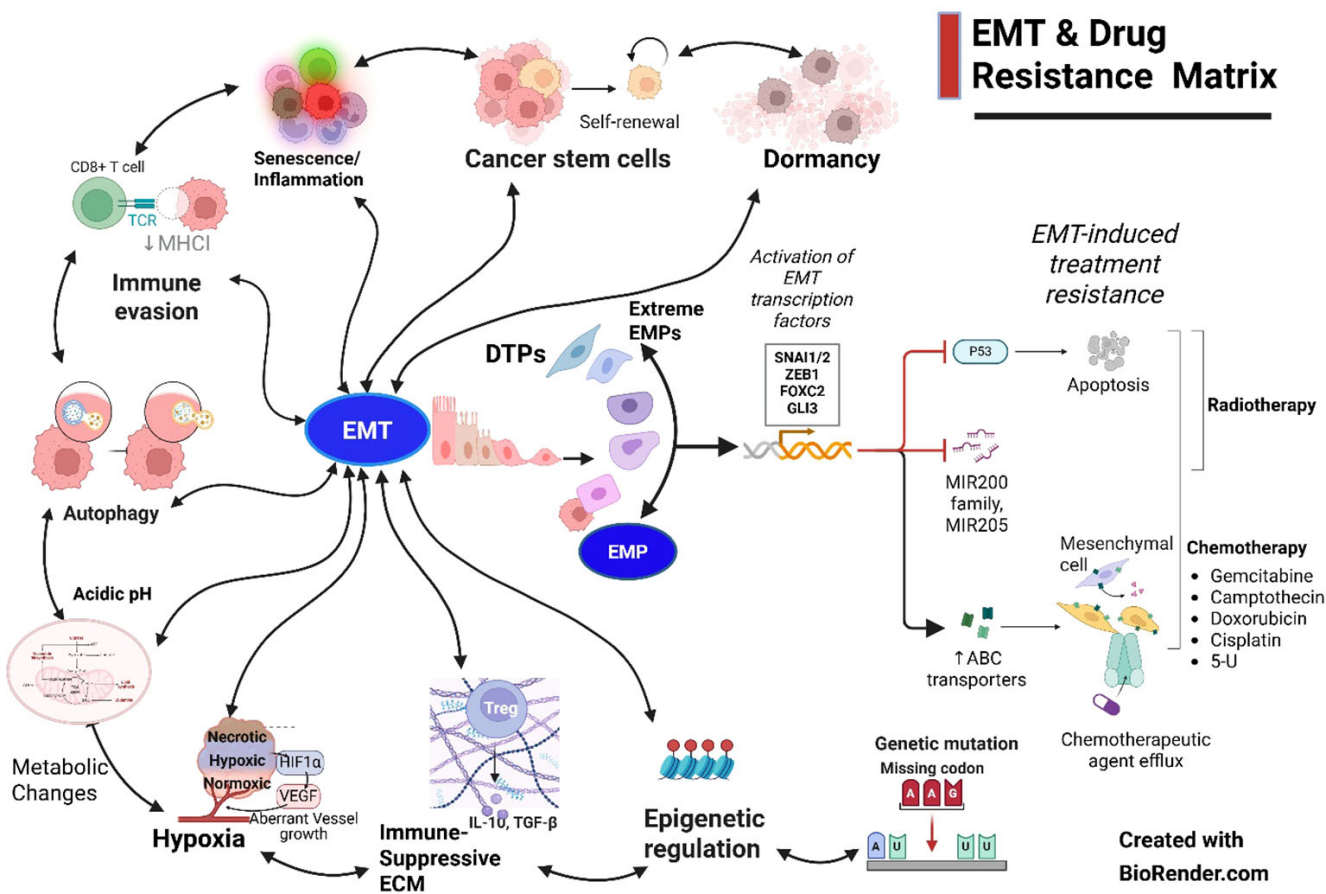
Cancer heterogeneity matrix. The web of pathways and the major reasons for primary/secondary resistance. EMT and plasticity (EMP) remain embedded in and promote it. Created in BioRender. Swamy K (2026) https://BioRender.com/blqfhrh.

Cancer cells hide within the compartmentalized TME, with diverse barriers that provide the basis for the development of multiple layers of resistance to therapeutic interventions (**Figure 5**). EMP, with its own multifaceted profile, is embedded in this complex, multifactorial network (**Figure 6**). Additionally, cancer cell-protective dynamic evolutions with unlimited mutational capacity form a web of therapy-escape-resistant mechanisms (**Figures 5**, **6**). This inhomogeneity can become more complicated with each unsuccessful line of therapy (2, 65).

In this highly resilient and dynamic cancer ecosystem, CSCs and EMT are tightly interconnected, with phenotypic switching. EMT can drive a non-stem cell state into a stem cell state, conferring drug resistance (66).

The architecture of this heterogeneity is spatial, functional (adaptability), and mechanistic. The hypoxic, acidic, and frequently necrotic tumor core is preferentially populated by quiescent, slow-cycling dormant cells and cancer stem cells, which are shielded from cytotoxic agents by poor perfusion. In contrast, the relatively well-vascularized/normoxic invasive edge harbors rapidly dividing, comparatively chemo- and radio-sensitive cells. Because drugs/cytotoxic-cell therapy delivery, oxygenation, and immune infiltration differ sharply between these compartments, the CTS protocol is deliberately engineered to address them in sequence: a). vascular normalization and antiangiogenic priming (Phase I) first improve perfusion and oxygenation of the core; b). MET-inducing epigenetic modifiers together with chemo-/radiotherapy (Phases I–II) then sensitize and debulk the proliferative edge; c). which unmasks and readies the tumor for immunotherapy (Phase III); d) maintenance/metronomic therapy, Cell therapies, and senolytic measures are timed to target DTPs (Phases IV–V). At phases III to V, pulsed/cyclical SBRT boosts of ~8 Gy per fraction (total dose determined by the toxicity profile and nearby organs at risk) can be a useful adjunct to address residual cancer cells and improve memory cell pool when indicated. Maintenance/metronomic therapy, Cell therapies, and senolytic measures (Phases IV–V) are timed to target resistant cancer cells/DTPs. Explicitly matching each therapeutic agent to the physical, functional, and mechanism-based zone it can penetrate and act effectively is therefore central to bridging tumor heterogeneity and pharmacological delivery (7, 67).

## 5 Targeting tumor heterogeneity and the EMP maze

**Figure 7:**
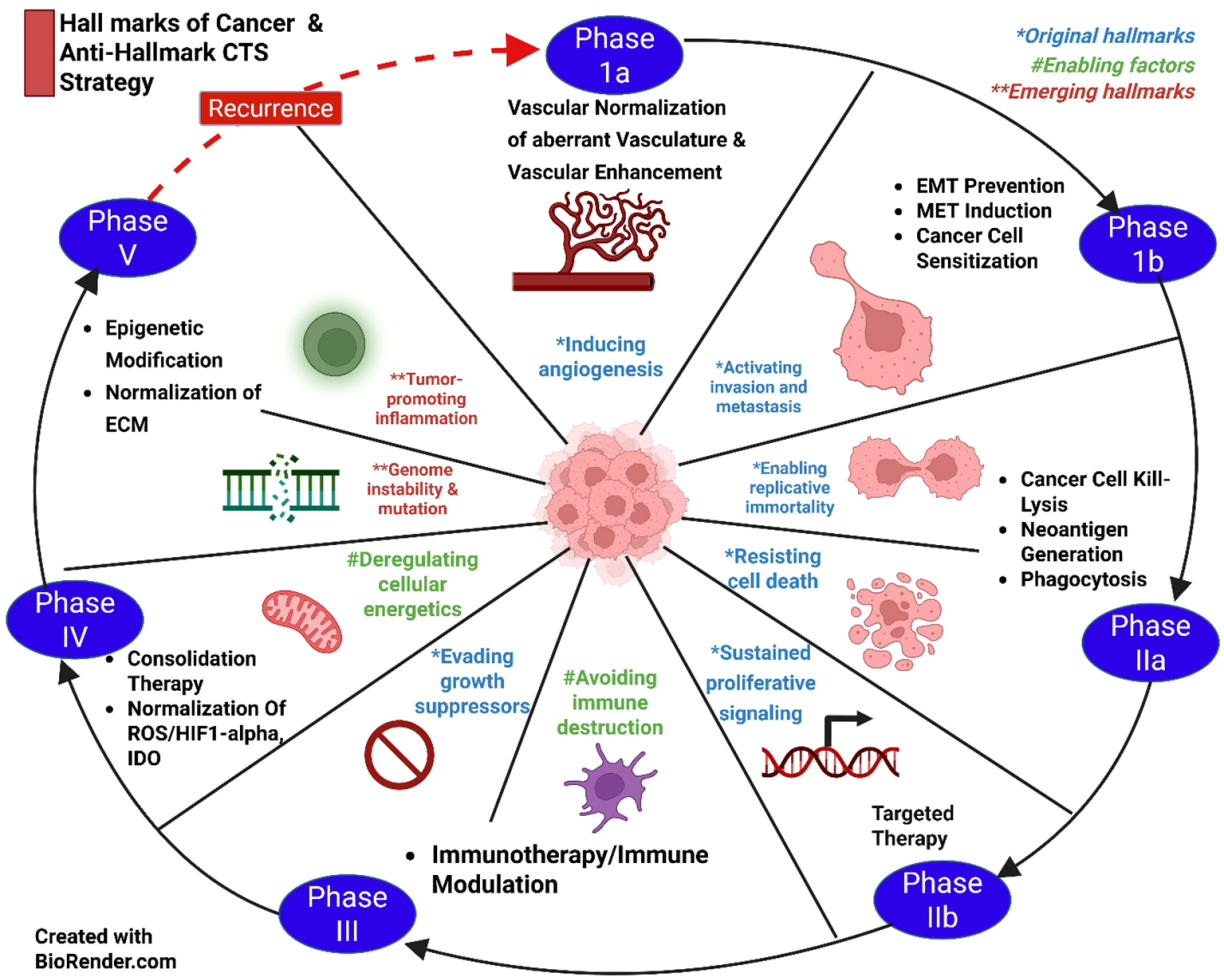
Principles of anti-hallmark targeting mechanisms of heterogeneity and EMP resistance network. Created in BioRender. Swamy K (2026) https://BioRender.com/z0ik8o2.

**Figure 8:**
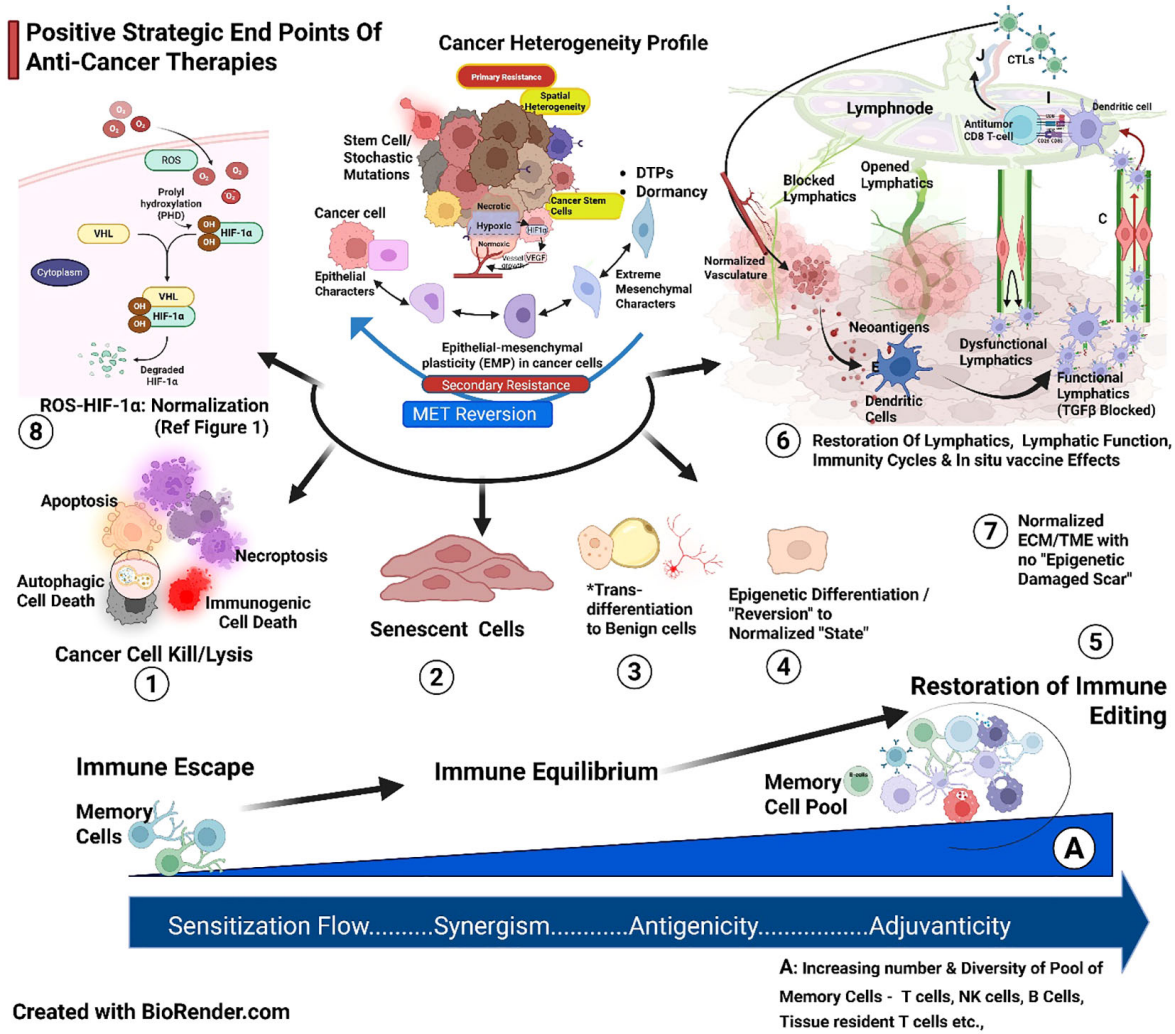
Strategic end points for successful anticancer therapies. Phenomena 1–8 present the steps toward a molecular cure for cancer. Focusing on (A) represents an increase in *in situ* effects, driven by improvements in the memory cell pool, primarily through antigenicity and adjuvanticity, restoring immune editing, preventing recurrence, and achieving long-term control. Created in BioRender. Swamy K (2026) https://BioRender.com/0125y0q.

The dynamic interplay between EMT, which promotes metastasis, and MET, which drives proliferation, represents a double-bind situation yet a therapeutic opportunity for an advanced, adaptive approach (68). This biological necessity provides a compelling therapeutic rationale: pharmacologically inducing MET can reprogram aggressive, mesenchymal-like cancer cells to revert to a more differentiated, less motile, and therapeutically susceptible epithelial state primed for timed chemosensitization (69). The deepest evasive action and the most difficult to eliminate come from drug-tolerant persisters (DTPs), which have diverse resistance mechanisms and await the appropriate microenvironmental conditions to re-proliferate (40, 70), long after standard therapy ends and presumed cure is achieved. Instead, the pleiotropic, transitioning, and interconversion unstable states should be used to drive them toward terminal (trans)differentiation into adipocytes or toward apoptosis, as demonstrated in breast and pancreatic cancers, respectively (6, 13, 36), using a clear therapeutic window (71) (**Figures 7**, **8**).

Recent advances in incorporating epigenetic modifiers into treatment protocols could help break this impasse. Appropriate non-cross-resistant combination therapies within a complementary CTS strategy, guided by minimally invasive monitoring approaches such as plasma VEGF levels (72), exploit tumor-specific transitional states (EMP) using epigenetic modifiers, state-of-the-art technologies, and tumor micro- and macro-environmental immunity-inducing factors. Histone deacetylase inhibitors (HDACis) can reprogram gene expression to favor an epithelial state (73–75). Advanced artificial intelligence and nanotechnology hold considerable potential to accelerate this approach. Kwon et al. (2021) also detail the “right” timing, combination, sequencing, and delivery of immunotherapy in combination with other treatments, favoring immunotherapy as a priming therapy preceding other anticancer therapies (76).

### 5.1 Epigenetic modifiers

#### 5.1.1 Eribulin—a clinical proof-of-concept for use of epigenetic modifiers

The most definitive clinical validation of therapeutic MET induction comes from studies of eribulin mesylate, a Food and Drug Administration (FDA)-approved microtubule dynamics/chromatin remodeling inhibitor used in metastatic breast cancer. Beyond its primary antimitotic function, eribulin possesses a unique, non-mitotic mechanism of action that reverses EMT, normalizes vasculature, reverses tissue hypoxia, and remodels the ECM (77, 78). Preclinical work in triple-negative breast cancer (TNBC) models and patient-derived orthotopic xenograft (PDOX) models demonstrated that eribulin causes a profound phenotypic shift from a mesenchymal to an epithelial state, characterized by the upregulation of epithelial markers (such as E-cadherin) and the downregulation of mesenchymal markers (including vimentin and the key EMT transcription factor ZEB1). To manage the EMT-MET double bind, eribulin can be used as pre-priming therapy before the emergence of chemoresistance and metastatic progression in breast cancer (79, 80). At the mechanistic level, this MET-inducing activity is increasingly recognized as an epigenetic, non-mitotic effect rather than a consequence of microtubule inhibition alone. Eribulin drives ZEB1–SWI/SNF-directed chromatin remodeling by disrupting the ZEB1-dependent recruitment of the SWI/SNF (BRG1) chromatin-remodeling complex that normally represses epithelial genes such as E-cadherin. In this process, it reorganizes the chromatin landscape toward an epithelial transcriptional program (69, 73). Critically, in the treatment-naïve setting, eribulin induces MET in primary triple-negative breast cancer in patients, whereas conventional taxane-based chemotherapy does not. This MET induction by Eribulin does not happen after acquired resistance to other agents develops. Consequently, this chromatin reprogramming in naïve resistant cells renders them sensitized to subsequent chemotherapy (69). These patient-level findings reposition eribulin from a purely theoretical pre-priming epigenetic sensitizer to a clinically applicable one and support its early, treatment-naive deployment within Phase Ib of the CTS protocol (69).

#### 5.1.2 Other epigenetic modifiers

Direct EMT-TF inhibitors restore p53 function in preclinical models harboring K-Ras mutations (81). Other compounds, such as Co(III)-Ebox, prevent Snail1 from binding to the E-cadherin promoter, thereby blocking its primary repressive function (82). In pancreatic adenocarcinoma, suppression of EMT with the nontoxic, low-dose ML210 chemical compound, in combination with gemcitabine, prevented cell migration (83). In an animal study, systemically administered nanoparticle therapy that releases siDANCR after endosomal escape at low pH degrades cellular DANCR via the RNA-induced silencing complex (RISC), thereby preventing EMT and phosphorylation (84). Repurposed agents, such as metformin, inhibit EMT via mammalian target of rapamycin complex (mTORC)/TGF-β signaling (85). Salinomycin, as an AMP-activated protein kinase (AMPK) inhibitor, reverses doxorubicin-induced EMT by upregulating E-cadherin and can induce mitochondrial dysfunction and autophagy (86, 87). c-MET inhibitors such as crizotinib resensitize small-cell lung cancer cells to CT by blocking EMT (88). Restoring miR-200 function using therapeutic mimics can induce MET, upregulate E-cadherin, and reduce motility (48). These agents, their molecular targets, mechanisms of action, and stage of evaluation are summarized in **Table 1**.

**Table 1:** Representative epigenetic and EMT/MET-modulating agents discussed in Section 5.1.2, their molecular targets, mechanisms of action, and stage of evaluation.

Targeting drug	Target/pathway	Mechanism of action	Stage of evaluation
Snail–p53 binding inhibitors	Snail; p53	Restore p53 function and pro-apoptotic signaling in K-Ras-mutated cells (81)	Preclinical (81)
Co(III)-Ebox conjugate	Snail1; E-cadherin promoter	Prevents Snail1 binding to the E-cadherin promoter, blocking its repressive function (82)	Preclinical (breast) (82)
ML210	EMT suppression (GPX4); with gemcitabine	Low-dose, nontoxic EMT suppression; combined with gemcitabine prevents PDAC cell migration (83)	Preclinical (PDAC) (83)
siDANCR nanoparticle	lncRNA DANCR (via RISC)	pH-triggered endosomal release; RISC-mediated DANCR degradation; prevents EMT (84)	Preclinical (animal, TNBC) (84)
Metformin (repurposed)	mTORC/TGF-β	Inhibits TGF-β-induced EMT via mTORC/TGF-β signaling (85)	Repurposed; widely used clinically (85)
Salinomycin	AMPK; β-catenin/Wnt	Reverses doxorubicin-induced EMT, upregulates E-cadherin, induces mitochondrial dysfunction and autophagy (86, 87)	Preclinical (86, 87)
Crizotinib (c-MET inhibitor)	c-MET	Blocks EMT and resensitizes small-cell lung cancer to chemotherapy (88)	Approved (other indications); preclinical here (88)
miR-200 mimics	ZEB/miR-200 axis	Restore miR-200 function, induce MET, upregulate E-cadherin, reduce motility (48)	Preclinical (48)

#### 5.1.3 Mechanism-based classification of epigenetic modifiers

A growing body of preclinical and clinical evidence has identified several pharmacological agents that can be categorized under five categories: (1) agents that prevent EMT and invasive ability (68); (2) those that sensitize cancer cells through MET conversion for subsequent CT/RT/targeted therapy/immunotherapy, requiring proper timing and sequencing (79, 80); (3) agents that push cancer cells to a susceptible EMT/EMP state or lethal ROS when the cancer cells dwell in DTP, senescence, or dormancy states (89); (4) agents that keep cancer cells “blocked” in a cell cycle phase away from resistant phenotype evolution (10); and (5) the least explored category, reverting cells to a normalized, evolutionarily suppressed, relatively stable, or benign (trans)differentiation state (10, 40). This mechanism-based classification emphasizes where epigenetic modifiers should be integrated into the CTS strategy. The five categories, their molecular rationale, representative agents (e.g., eribulin, HDAC inhibitors, metformin), and recommended timing within the CTS framework are consolidated in **Table 2**.

**Table 2:** Mechanism-based classification of epigenetic modifiers (section 5.1.3), with molecular rationale, representative agents, and recommended timing/sequencing within the anti-hallmark CTS framework.

Category	Molecular target/rationale	Representative agents	Recommended timing/sequencing in CTS
(1) Prevent EMT and invasive ability	EMT transcription factors; invasion programs	EMT-TF inhibitors; vactosertib (TGF-β/ALK5) (68)	Pre-priming (Phase Ib) (68)
(2) Sensitize via MET conversion for subsequent CT/RT/targeted/immunotherapy	ZEB1–SWI/SNF chromatin remodeling; E-cadherin restoration	Eribulin; HDAC inhibitors (e.g., mocetinostat) (79, 80)	Pre-priming, immediately before CT/RT/SBRT/IO (Phase Ib → II) (79, 80)
(3) Push DTP/senescent/dormant cells to a susceptible EMT/EMP state or lethal ROS	BET bromodomain; ROS induction on cell-cycle re-entry	BET inhibitors (89)	Post-primary/maintenance (Phase IV) (89)
(4) Block cells in a cell-cycle phase away from resistant evolution	Cell-cycle checkpoints	Cell-cycle-arresting epigenetic agents (10)	Maintenance (Phase IV) (10)
(5) Revert to a normalized, stable, or benign (trans)differentiation state	Differentiation programs (e.g., PPARγ)	Differentiation inducers (rosiglitazone + MEK inhibitor); metformin (10, 13, 40)	Consolidation (Phases IV–V) (10, 13, 40)

### 5.2 Two concepts for one strategy

The first conventional concept and foundation of modern anticancer therapy is to eliminate as many cancer cells as possible as quickly as possible, to maximize the chance of a cure. Cancer therapies are administered at the maximum tolerated dose (MTD), a foundational concept in phase I clinical trials. The two premises of MTD approaches are: to eliminate cancer cells before resistance develops, and that resistant mutations are likely to arise from a large rather than a small population of cancer cells (40, 41).

According to the second concept, evolutionary-based therapy (EBT), the conventional MTD approach is the antithesis of Darwinian evolutionary dynamics. In this proposal, the resistance trait is already encoded as partial resistance at a low frequency in a small subpopulation. When it repopulates after MTD, it progresses into a whole resistance population. Currently, EBT is in the exploratory phase, focused on monitoring the proliferation of a small resistant population through dose adjustments and epigenetic marker monitoring as part of a dynamic treatment process. In advanced cancers, this approach maintains good quality of life without toxicity, and no attempt is made to eradicate all cancer cells (40, 41).

The following perspectives aim to unify these two concepts, based on the understanding that, in principle, they are not mutually exclusive but are two dimensions of the complex heterogeneity of cancer.

Operationally, unifying these concepts requires a defined trigger for transitioning a patient from the maximum-tolerated-dose (MTD) debulking of Phase II to the evolution-guided maintenance of Phase IV. We propose that this transition be governed not by fixed calendar scheduling but by quantitative minimal residual disease (MRD) assessment—using circulating tumor DNA, plasma VEGF kinetics, and emerging epigenetic and immunopeptidomic readouts. Evolution-based therapy is initiated once cytotoxic debulking reaches a measurable plateau and before a resistant subpopulation can repopulate. Within this maintenance phase, “adaptive therapy” algorithms, in which drug doses are actively modulated according to real-time tumor volume dynamics. The objective is to exploit competition between drug-sensitive and drug-resistant dormant cell types to prevent the emergence of resistance. This will also provide an opportunity for reactivated immune surveillance to eliminate persistent residual cancer cells. In a pilot clinical trial in metastatic castration-resistant prostate cancer, such tumor dynamics-guided dosing extended the median time to progression while substantially reducing cumulative drug exposure relative to standard continuous dosing, providing proof of principle for coupling adaptive scheduling into the CTS framework (40, 90).

## 6 Hypothesis—anti-hallmark combinations, timing, and sequencing strategy

Hypothesis One: In cancer therapy, normalization of the vasculature and TME is the sine qua non for the predictability of cancer elimination.

Hypothesis Two: Cancer therapy resistance is mediated by tumor heterogeneity, along with EMP, and can be overcome through a well-structured anti-hallmark CTS strategy. Additionally, this strategy should deactivate immune-escape signaling in the TME/ECM and restore immune editing by invoking cascading virtuous cycles of antigenicity and adjuvanticity.

It has been more than 26 years since the introduction of the hallmarks of cancer by Hanahan and Weinberg in January 2000 (91). They enumerated that tumorigenesis is a multistep process that ultimately culminates in a malignant phenotype, yet it can be simplified to six biological capabilities. Reiterating their concept in 2011 (92), they foresaw cancer research as an increasingly logical science, in which myriad phenotypic complexities are manifestations of a small set of underlying organizing principles. Revisiting these principles, we have designed a hypothesis to counter this multistep process using a systematic multi-phase protocol, proposed as an anti-hallmark (hallmark-degradation) hypothesis (**Figure 7**). **Table 3** depicts the proposed protocol matching the anti-hallmark concept.

**Table 3:** Schema of anti-hallmark combinations, timing, and sequencing protocol.

Phase	Strategy & mechanisms	Timing	Objective	Key references
Phase Ia	Pre-priming with Vascular Normalization: eg., AAGs (3 weeks on, 3 weeks off for extended normalization – Proposed - Yet to be validated */$)	Day 0	VN is essential for effective chemo-immunotherapy delivery; it improves oxygenation and TME, thereby increasing sensitivity to CT/RT/SBRT.	(95–98) Yang T et al.; Liu Z et al.; Magnussen AL & Mills IG; Guo Z et al. (101, 133) Jain RK; Goel S et al.
Phase Ib	Pre-priming: Epigenetic modifiers as CT/RT sensitizer and MET inducers	Starts at week 1 of AAGs (>Day 2)**	Reverse epigenetic changes; prevent EMT/stemness; sensitize cancer cells to subsequent CT/RT/SBRT.	(46) Guo H et al. (69); Bagheri M et al. (4); Huang Y et al. (74, 75); Schiedlauske K et al.; Meidhof S et al. (79, 80); Yoshida T; Lim HI et al. (108); Park J et al.
Phase II	Priming therapy: MTD strategy, cytotoxic therapy, CTLs activation, professional phagocytosis, decreased ISP, *in situ* vaccination, nanoparticle therapy, concurrent epigenetic therapy	Start CT/RT (SBRT) Day >Day 2 of the start of Phase I	Cancer cell lysis (preferably by ICD); neoantigen generation and restoration of immunity cycles; immune-suppressive cell depletion in TME; enhanced normalized vascularity; improve lymphatic *function* with TGF-β blockers.	EMP prevention (44, 102): Frey P et al.; Celià-Terrassa T et al. (106); Srinivasan et al. (111–113); Phagocytosis/ISP (72); Hughes CS et al. (110); Liu N et al. (101) Goel S et al.
Phase III	Primary therapy: Optimizing targeted/immunotherapy; timed/pulsed SBRT boost; immune adjuvants; phagocytosis checkpoints; *in situ* vaccination; nanoparticle therapy; epigenetic therapy	2–3 weeks after neoadjuvant CT/RT (SBRT) or after surgery	Stage set for optimizing response to immunotherapy; cancer cells “unmasked” and TME-modulated for effective drug concentrations; mechanotransduction barriers depleted; TME reset with active CTLs; integrated SBRT boost for dynamic *in situ* vaccination effect	(142) Sordo-Bahamonde et al. (143); Lussier DM et al. (139–141); Breen WG; He K; Spaas M et al. (114); Feng K et al. (110); Joshi S & Durden DL
Phase IV	Post-primary therapy: Eliminating MRD/dormancy/DTPs/senescence; consolidation with immunotherapy; anti-evolutionary resistance; BET inhibitors; normalized soft ECM	2–3 weeks after primary therapy; maintenance as per guidelines	Design maintenance therapy with the least long-term side effects; develop long-term anticancer/repurposed drugs to eliminate dormancy & prevent recurrence; increase adoption of epigenetic modifiers	(39, 151) Zhao Y et al.; Zhang M et al. (6, 59); Lu W & Kang Y; Williams ED et al. (89); Chen M et al. (10); Pensotti A et al. (72); Hughes CS et al.
Phase V	Probative/consolidation therapy: Abate inflammation; mitigate ECM “epigenetic damage scar”; restore immune editing; ECM reprogramming; to prevent late recurrences	From the point of apparent cure or unacceptable toxicity	Maintain supple ECM; Normalize HIF-1α -TGF-β- ROS axis; Reduce inflammation via senolytics/lifestyle; Advanced molecular monitoring for early detection of molecular recurrence	(89) Chen M et al. (41); Škarková A et al. (119, 120); Short S; Carpenter et al. (122); Kirkland JL & Tchkonia T (149); Marino et al. (40); Gatenby & Brown (72, 145); Hughes CS et al.; Hickman et al.

**Figure 9:**
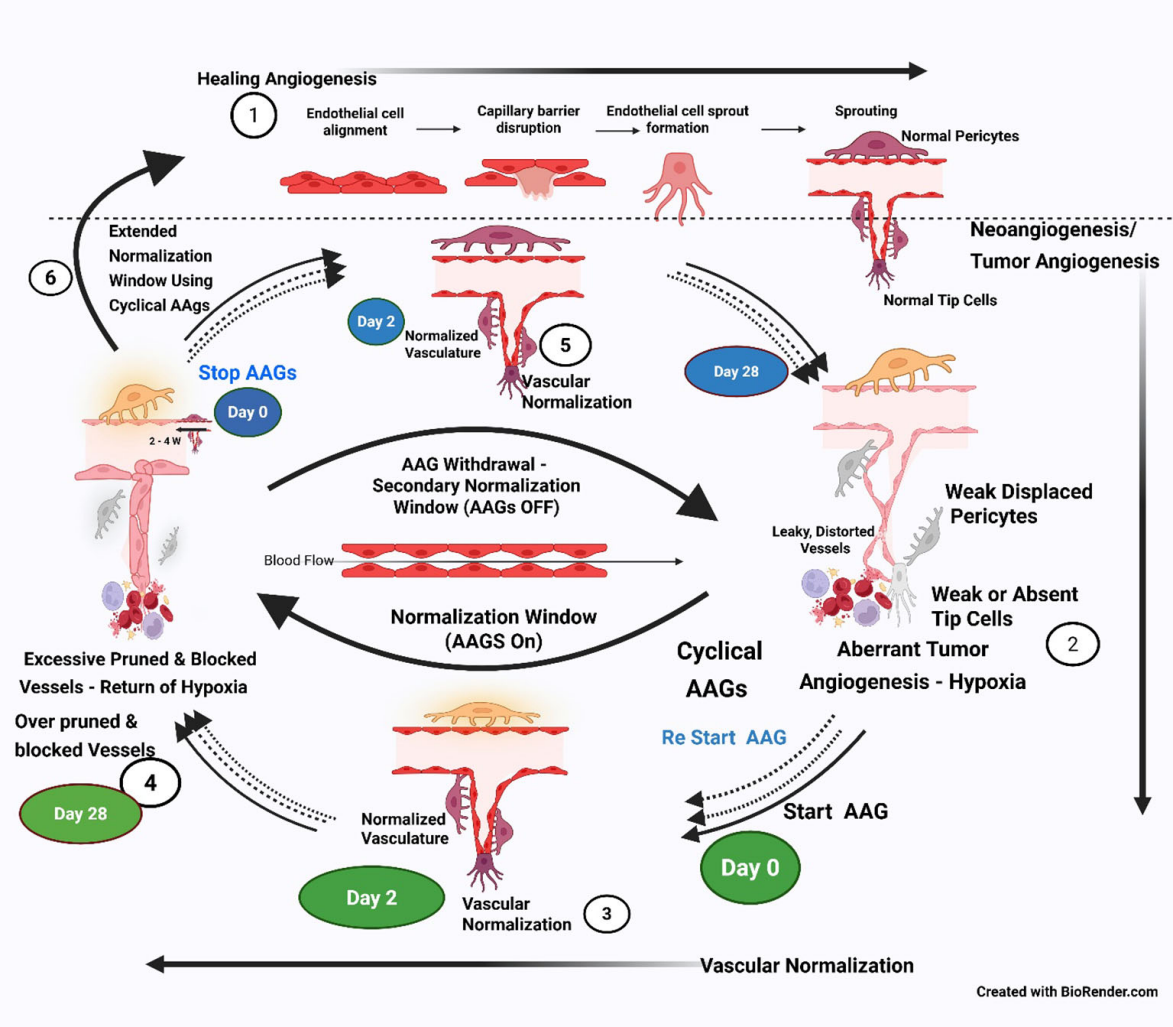
Extended vascular normalization concept. Cyclical AAG dosage: 3 weeks on and 3 weeks off, creating extended/multiple normalization windows. This is a proposed concept rather than a validated schedule. It is essential to note that the drug-free interval for antiangiogenics carries a risk of *rebound angiogenesis* and must be biomarker-guided (DCE-US/plasma VEGF) and tailored to the drug-specific kinetics (monoclonal antibody vs TKI) (99). The accruing data will indicate the optimal on-and-off schedule for the individual drug and tumor type/site. As a baseline, the 3 weeks on, 3 weeks off schedule is based on the publication by Goel et al., 2011 (101), in which the authors reported that initial pruning of immature vessels with vascular normalization leads to excessive pruning by Day 28, with increased hypoxia. The Magnetic Resonance Imaging changes of the 2^nd^ window of vascular normalization returned after recommencement of toxicity-induced drug holidays. There is no further data indicating that such cycles can be repeated to open multiple normalization windows. (Due to the tumor growth timeline and short lifespan, animal studies would be challenging) Created in BioRender. Swamy K (2026) https://BioRender.com/6p85x2g.

AAGs, anti-angiogenic agents; VN, vascular normalization; BET, bromodomain and extraterminal domain; CCME, cancer cell microenvironment; CT, chemotherapy; CTL, cytotoxic T lymphocyte; ICD, Immunogenic Cell Death; DTPs, drug-tolerant persister cells; ECM, extracellular matrix; EMP, epithelial-mesenchymal plasticity; EMT, epithelial-mesenchymal transition; HIF-1α, hypoxia-inducible factor-1α; ICS, intracellular space; ISP, interstitial pressure; MET, mesenchymal-epithelial transition; MRD, minimal residual disease; ROS, reactive oxygen species; RT, radiotherapy; SBRT, stereotactic body radiotherapy; TGF-β, transforming growth factor-beta; TILs, tumor-infiltrating lymphocytes; TME, tumor microenvironment. *Proposed, needs to be validated. See **Figure 9**. ^$^A low dose continuous AAGs protocol can be considered with neoangiogenesis imaging and molecular monitoring. **This aligns, at the minimum, with the transient vascular normalization window (~days 2–8) to maximize penetration of the epigenetic/MET-inducing agent (Phase Ib) into the tumor core, thereby achieving synergistic effects, which is to be validated.

Hypothesis One and related aspects are detailed elsewhere (93). Hypothesis Two is addressed in the present article, based on the mechanism that counters the specific hallmark of cancer (anti-hallmark mechanisms), thereby helping to place any treatment (present or future) in a particular phase of the CTS strategy. The heterogeneity across cell populations in various phases of cancer hallmarks necessitates the use of non-cross-resistant combinations at safe, optimal doses (94). The eight strategic endpoints for this strategy’s success are enumerated in **Figure 8**.

### 6.1 The prerequisite—countering tumor neoangiogenesis and hypoxia

Due to deficient and aberrant vasculature, increased interstitial pressure, and mechanotransduction barriers, effective drug delivery into all three compartments—the cancer TME, the cancer cell microenvironment (CCME, intercellular space), and the intracellular space (ICS)—is hindered (7, 76). This patchy distribution of effective drug concentrations results in suboptimal response and invites the evolution of resistant pathways. For example, taxanes, which bind to β-tubulin, and anthracyclines, which interact with topoisomerase II, require effective concentrations at their target sites to overcome efflux mediated by ATP-binding cassette (ABC) transporters that reduce intracellular drug levels (94). Additionally, loss of stemness from anti-EMT therapy reduces ABC protein expression, thereby reducing drug efflux and improving CT efficacy (4).

Several publications highlight the significance of vascular normalization as a precondition for integrating it with other established anticancer therapies, including immunotherapy (95–98). The theoretical concept of extending vascular normalization using anti-angiogenic agents (AAGs) is shown in **Figure 9**, and its optimization requires guidance from imaging or plasma VEGF levels. This proposal of a cyclical, three-weeks-on/three-weeks-off schedule to extend the normalization window or to have multiple normalization windows must, however, be interpreted with explicit caution rather than presented as an established effect. Preclinical evidence demonstrates that the tumor vasculature can regrow rapidly once anti-angiogenic pressure is withdrawn: empty basement membrane sleeves and surviving pericytes act as a scaffold for endothelial sprouting within 1 day, with essentially full neovascularization within approximately 7 days of drug cessation (99). Drug holidays therefore carry a genuine risk of rebound neoangiogenesis and accelerated regrowth, so the presumed gain in normalization during the “off” period should be regarded as a hypothesis requiring prospective validation. The kinetics are, moreover, drug-dependent: the slow clearance and sustained target engagement of monoclonal antibodies such as bevacizumab yield a different normalization timeline than the rapid on/off pharmacodynamics of small-molecule tyrosine kinase inhibitors. The timing of the CTS sequence must accordingly be tailored to the specific drug selected. The timing strategy should be guided by real-time, patient-specific biomarkers—such as dynamic contrast-enhanced ultrasound (DCE-US) perfusion and blood volume parameters or plasma VEGF kinetic monitoring—to identify the closure of the normalization window and to preempt the onset of rebound angiogenesis on an individualized basis (72, 100).

### 6.2 Disabling invasion, replicative immortality, and cell death resistance: adapting the principle of “cancer cell therapy sensitization flow”

In the planned sequencing, the targeted drug and epigenetic modifiers that tend to sensitize subsequent therapy should be placed in the earlier phase of the protocol. Mesenchymal cells are sensitive to the AXL inhibitor SGI-7079. The TGF-β inhibitor SB-431542 or receptor antibodies increase sensitivity to carboplatin. An EMT signature showed better control with erlotinib in non-small cell lung cancer (NSCLC) but not in other therapies. EMP showed resistance to EGFR and PI3K/protein kinase B (AKT) inhibitors, irrespective of mutational status. However, when CT follows these inhibitors, it demonstrates greater sensitivity than when either is used alone in the presence of EMP (102).

Standard anticancer treatments, such as CT, targeted therapy, and radiation, can promote oncogenic type III EMT, leading to immune evasion and anoikis resistance via Snail- and Slug-mediated inhibition of p53-induced apoptosis (103–105). After CT/RT, plasticity and DTPs are believed to constitute a reservoir that eventually leads to irreversible genetic mutations and a recurrent, resistant cancer cell population. RT is generally indicated in 50–70% of cancer patients and is known to induce EMT through the TGF-β, Notch, and extracellular signal-regulated kinase (ERK) pathways (106). RT-induced EMT can be overcome with timed epigenetic modifiers. In a phase I study of nasopharyngeal carcinoma, combining EGFR tyrosine kinase inhibitors (TKIs) with intensity-modulated RT (Trial NCT01534585) demonstrated feasibility and clinical activity (107). Vactosertib, a TGF-β/ALK5 inhibitor, reduces oxidative stress, fibrosis, and stemness, thereby inhibiting EMT, and also exerts an independent antitumor effect in breast cancer (108). The DNA methyltransferase (DNMT) inhibitor decitabine sensitized cancer cells to RT and resensitized ovarian cancer cells to cisplatin/carboplatin. Bromodomain protein 4 (BRD4) inhibitors enhance sensitivity to chemo-RT and prevent RT-induced PD-L1 upregulation in NSCLC (109). The synergistic effect of the multimodal approach, combined with advances in nanomaterial-based *in situ* vaccination technology, awaits mechanistic integration into clinical practice (110).

The phagocytosis checkpoint in immunotherapy, which overcomes “don’t eat me” signals from cancer cells, complements T-cell responses (7, 111–114).

### 6.3 Managing sustained proliferative signaling and immune destruction avoidance

Cancer cells employ several mechanisms to evade targeting and immune destruction. One is the transdifferentiation of malignant phenotypes to different embryonic components. In prostate cancer, the development of a hormone-independent neuroendocrine phenotype can be prevented by anti-IL-6 monoclonal antibody siltuximab and JAK kinase inhibitor ruxolitinib, laying the strategic foundation for intercepting EMP at junctional points. Transformed small-cell lung cancers (SCLCs) in NSCLC respond better to taxanes than *de novo* ones and are resistant to immunotherapy (115). DTPs require transitioning from a predominantly MTD schedule to a maintenance schedule complemented with targeted epigenetic reversion therapy. DTPs are vulnerable to bromodomain and extraterminal domain (BET) protein inhibitors, especially when re-entering the cell cycle, which induces lethal levels of ROS-induced apoptosis (89). Other mechanisms include dedifferentiation/undifferentiation, in which the cells become more aggressive and less responsive to immunotherapy (116). Conversely, epigenetic forward differentiation of cancer cells terminates malignant proliferation signaling (**Figure 8**) (14).

### 6.4 Prevention of genome instability, reduction of tumor-promoting inflammation, and restoring immune surveillance

Any drugs/treatments in the flow must not leave a genomically unstable “epigenetic damage scar.” Altered epigenetic marks can influence inheritable transcriptional effects on surrounding genes. These changes can derail cell identities and could explain late recurrences, making this field a fascinating area for further study. A related phenomenon is therapy-induced stromal senescence, which can induce inflammation in fibroblasts (12, 117).

A cure is feasible with the MTD approach in the patient group in which resistant traits have not yet been established. In other cases, phenotypic resistance can evolve during MTD. The current article proposes a CTS strategy that combines both MTD and evolutionary principles to preempt cancer cell resistance. The importance of long-term antitumor defense in the simultaneous multi-hit tripartite approach—which reduces tumor load, increases immunogenicity, and reverses immunosuppression—is documented in the literature (118). Epigenetic modifications to reduce inflammation and recurrence can be explored using senolytics/senostatics, although their clinical effectiveness remains unestablished (119, 120). Also, caution is essential because therapy-induced senescence is a double-edged outcome: senescent cells elaborate a senescence-associated secretory phenotype (SASP)—a context-dependent milieu of pro-inflammatory cytokines, chemokines, and proteases—that can paradoxically promote proliferation, EMT, angiogenesis, and immunosuppression in neighboring cells and can reawaken dormant drug-tolerant persisters (121). Consequently, when senescent cells are among the products of the CTS strategy, targeted depletion of these senescent cells with discoverable, effective senolytics [the current evidence for the utility of existing senolytics is questioned (120)] during the maintenance phase ensures that the SASP cannot rescue surviving DTPs. An intermittent “hit-and-run” senolytic schedule is rational because senescent cells take weeks to reaccumulate after being cleared, significantly reducing the risk of toxicities (122).

This strategy targets the resistant cell population, one after another, in a systematic protocol, with equal focus on epigenetic reversion, which serves as the backbone of the therapeutic schedule (**Figure 7**). Simultaneously, downstream oncogenic drivers are eliminated through the logical sequencing of trimodal CT, RT, or immunotherapy along with targeted therapy (115). The present protocol design simultaneously enhances the immune cycle and the pool of memory cells, restoring/resetting immune editing.

**Figure 10:**
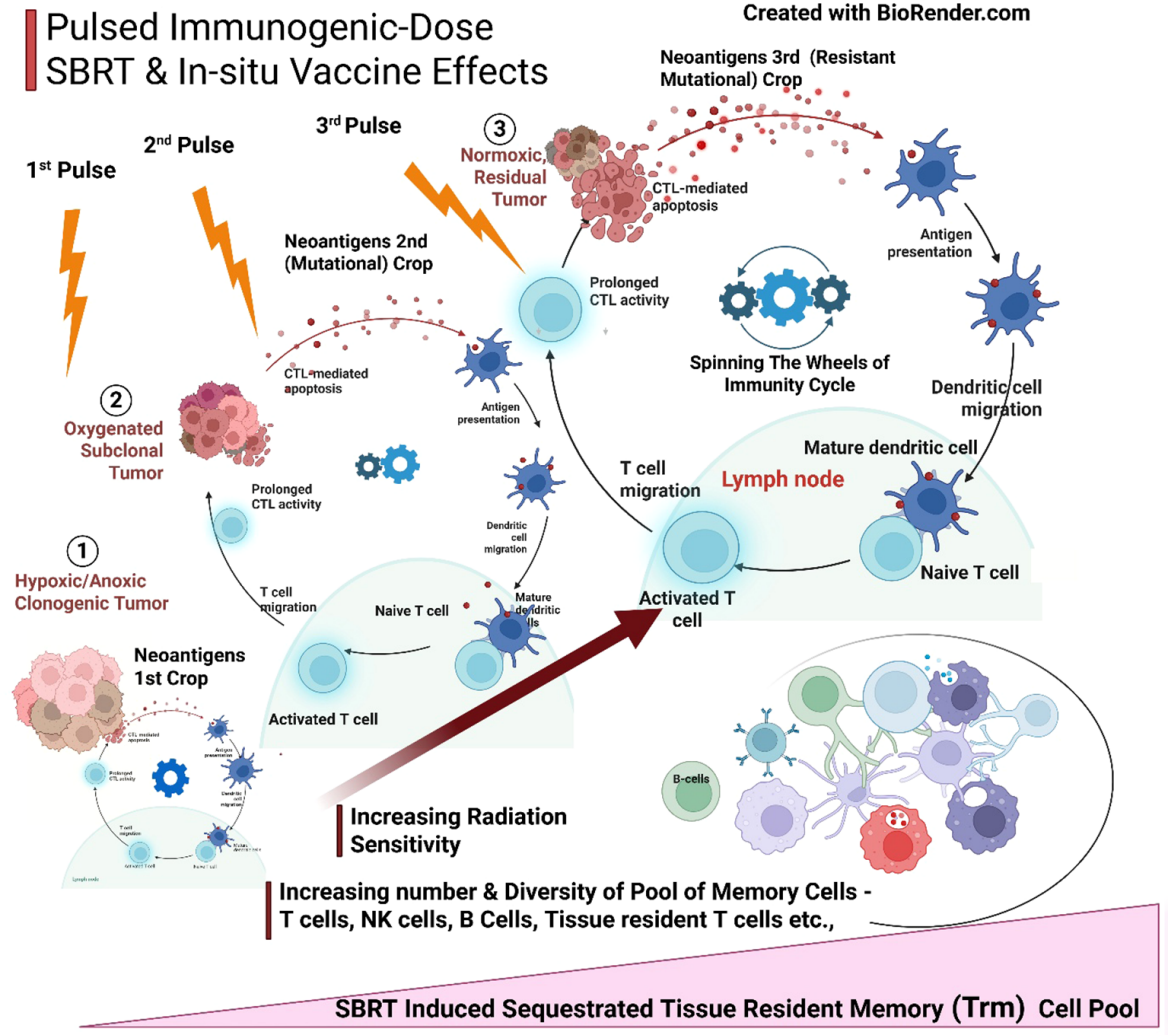
Figurative representation of *in situ* vaccination using pulsed/boost immunogenic dose SBRT. Pulsed SBRT (two or more) optimizes immune cycles, generating an expanding repertoire of neoantigens, tumor-specific T cells, and memory cells to be synchronized with immunotherapy (7, 142). Created in BioRender. Swamy K (2026) https://BioRender.com/84wka6v.

ROS, HIF-1α, and TGF-β are the three primary theoretical targets. However, these also play crucial roles in normal body homeostasis, requiring fine targeting. Surgical excision of residual lesions or pulsed/cyclical SBRT boost should be timed to remove/eliminate core DTPs, dormant, and stem cells (**Figure 10**).

### 6.5 Drugs with multilevel broad-spectrum anti-hallmark action

The critical role of drugs acting at multiple levels of the pathways—e.g., eribulin mesylate as a MET inducer, chemosensitizer, and vascular promoter, or TGF-β blockers with immune-enhancing, anti-inflammatory actions (e.g., vactosertib)—has excellent potential in the therapeutic landscape (79, 80, 108). Drugs with multispecific and secondary TGF-β-blocking actions would add value in preventing late recurrences (123). Valsartan, used routinely as an antihypertensive angiotensin II receptor blocker, has multiple levels of action and blocks TGF-β, angiotensin II receptor type 1 (ATR1), etc. It acts both as a chemotherapeutic sensitizer and reduces inflammation and ECM deposition, improving ECM–immune cross-talk and preventing fibrosis in animal models (124, 125). In a phase II trial, losartan, in combination with FOLFIRINOX, followed by chemoradiotherapy, was clinically beneficial in pancreatic cancer (7). Restoring “eat me” signals using phagocytosis checkpoint immunotherapy also has the potential to impact multiple hallmark levels (126). The biomechanical rationale for repurposing angiotensin-blockade drugs, which downregulates TGF-β signaling, deserves emphasis in densely desmoplastic solid tumors such as pancreatic ductal adenocarcinoma, where a stiff, collagen- and hyaluronan-rich stroma generates high solid stress and interstitial fluid pressure that collapse the microvasculature and mechanistically exclude drugs and immune effectors. Angiotensin II type 1 (AT1) receptor blockade uniquely targets this physical barrier by inhibiting cancer-associated fibroblast activation and TGF-β-driven matrix deposition. This decompresses the stroma, lowers interstitial fluid pressure, and reopens collapsed vessels, thereby improving perfusion, drug delivery, and the infiltration of cytotoxic T cells and immune therapy deployed in Phase III (7, 125).

### 6.6 Supporting literature evidence for the CTS strategy

This hypothesis diverges from conventional approaches by viewing tumor heterogeneity and EMP not as barriers to treatment but as targetable vulnerabilities when approached with an appropriate CTS protocol. The following supporting evidence exists:

CTS Synergistic Effects: Therapy combinations have evolved over the last 75 years, primarily due to synergistic effects of their mechanisms of action. Currently, these combinations are being increasingly tested in immunotherapy trials. TGF-β inhibitors, when combined with checkpoint inhibitors, simultaneously block EMT and improve immune cell infiltration (127).

Importance of Timing: In an *in vitro* study, prolonged pemetrexed pretreatment increased the persistence of cisplatin-induced DNA damage and reduced EMT-associated resistant cancer stem-like cell populations compared to concurrent treatment (5). Eribulin before standard CT sensitizes subsequent CT (79, 80). Establishing effective multidrug combinations, timing, and sequencing, followed by systematic risk-adapted stratification approaches in clinical trials for pediatric malignancies, is a classic example of controlling cancer and reducing toxicities (128).

Clinical Evidence: The NEJ009 trial showed a higher objective response rate (84%) than EGFR-TKI monotherapy (67%), with delayed resistance, manageable toxicity, and improved progression-free survival (PFS) in advanced (stage III-N2) EGFR-mutant NSCLC (129). Improved overall survival (OS) was observed with first-line osimertinib-CT compared with osimertinib alone in the updated phase III FLAURA2 trial, published in January 2026, and the increased incidence of adverse events was reversible (130). EGFR-mutant NSCLC is a highly specific, oncogene-driven paradigm, and the evolutionary pressures exerted by targeted tyrosine kinase inhibitors may differ markedly from those exerted by broad-spectrum cytotoxic chemotherapy, radiation, or immunotherapy regimens in other tumor types and sites. The upfront-combination principle, a first-of-its-kind (Global Phase III trial) with recently updated randomized trial results showing improved OS, supports the CTS rationale at the conceptual level as an example, without any intent to directly extrapolate or generalize across all anti-cancer therapy settings.

Vascular Normalization Versus Vascular Disruption with SBRT: A vascular normalization strategy with SBRT is strongly debated against the vascular disruption strategy. The listed criticisms of vascular normalization include: (a) the dynamicity and its window period make it challenging in clinical implementation, (b) it varies with tumor type, (c) patient-dependent intratumoral variation, (d) dose-dependency, and (e) the enigma of the “normalization window.” However, immunosuppressive signals, including the induction of the three-prime exonuclease 1 (TREX1) pathway, HIF-1α, TGF-β, IL-10, and myeloid suppressor cells, are recognized in the vascular disruption approach (131, 132), which may be a critical deciding factor in the present-day immunotherapy-oriented era.

The paradigm-changing concept of vascular normalization to retain the therapeutic sensitivity of cancer cells and deliver drugs at the cancer cell level, in contrast to the existing concept of vascular disruption-based cell killing (the superiority of one over the other is still being debated), was proposed in 2001 by Jain (133). Vascular normalization has special connotations with SBRT. Limiting the SBRT dose per fraction to 8–10 Gy spares endothelial integrity and, consequently, the ECM and the immune-suppressive island of “epigenetic scar”, which can become a recurrence-prone area (12). SBRT doses exceeding 10 Gy per fraction cause endothelial disruption and immune-suppressive ECM changes in a sarcoma model, which are unfavorable for long-term control (134). With the increasing role of immunotherapy across all cancers, the importance of avoiding epigenetic ECM scars supports a normalization approach retaining the immunogenic benefits of SBRT at 7–10 Gy per fraction. Improvement of immune cross-talk not only enhances the efficacy of immunotherapy but also reduces toxicity (135). The immunological rationale for capping the fraction size at 8–10 Gy is mechanistically grounded in the cytosolic DNA-sensing cGAS–STING pathway. At these immunogenic doses, radiation-induced double-stranded DNA that accumulates in the cytosol activates cGAS–STING and downstream type I interferon (IFN-β) secretion, driving immunogenic cell death and the maturation of Batf3-dependent dendritic cells that prime tumor-specific CD8+ T cells. Crucially, this immunogenicity is self-limiting at higher doses: the three-prime repair exonuclease 1 (TREX1) is induced once the dose per fraction exceeds approximately 10–12 Gy and degrades cytosolic DNA, thereby nullifying cGAS–STING signaling and the consequent dendritic cell priming (136). This provides the molecular basis for preferring fractions in the 7–10 Gy immunogenic range and for delivering them in a repeated/pulsed fashion. Moreover, SBRT-induced vascular normalization/enhancement acts synergistically with systemic immune checkpoint inhibitors—improving immune-vascular crosstalk, T-cell infiltration, and oxygenation. Thus, SBRT with a 7–10 Gy dose range vascular normalization/enhancement facilitates immunotherapy responsiveness primed by T-cell infiltration (135, 137).

Cyclical SBRT enhances the long-term “memory cell bank” to prevent recurrence, generating an *in situ* vaccine effect that can be complemented by *in vitro*/ex vivo or intratumoral vaccines (**Figure 10**) (137). Lesions that no longer respond to immunotherapy combinations may exhibit enhanced antitumor immunity through cancer cell kill, phagocytosis, decreased interstitial pressure, improved drug delivery, and re-oxygenation (7, 138). In the KEYNOTE-01 trial in NSCLC, even at a median of 9.5 months after RT, the immunotherapy group with a previous history of RT had longer progression-free and overall survival (139). Clinical benefits of SBRT (3 × 8 Gy) followed by pembrolizumab have been reported in patients with solitary lung metastases (139–141). Induction of immunogenic cell death improves tumor “hotness” and immune cell infiltrates (142). This immunogenic cell death dose range induces mutational neoantigens in the *in vivo* KP sarcoma model, sensitizing it to immunotherapy when the hot tumor replaces a cold tumor (143).

It is an opportunity to use a residual gross tumor as an *in situ* neoantigen provider by applying principles of antigenicity and adjuvanticity through local ablative therapies, including SBRT (**Figure 8**) (144), in select situations.

Advances in Monitoring Technology: Among all cytokines, the STARPAC phase I trial (NCT03307148) in pancreatic cancer indicated that plasma VEGF correlates with response early in the course of CT (72). Therefore, it is a promising approach that warrants further evaluation to support the routine adoption of the VEGF serum biomarker. Plasma immunopeptidome mining could make it a significant biomarker for follow-up monitoring and an ultra-specific therapy targeting tool in the coming years (145).

### 6.7 Methodology of implementation of CTS strategy

Based on the review above, the proposed strategy comprises five phases that must be implemented systematically. The first step is normalization of the vasculature, which will improve delivery of drugs/cytotoxic cells (to TME, CCME, and ICS) and reduce hypoxia, targeting the primary factor in the genesis of cancer. Other steps immediately follow (**Table 3**).

### 6.8 Example of trial protocol flow: breast cancer

The proposed anti-hallmark CTS protocol requires initiating therapy with AAGs. Bevacizumab, the VEGF monoclonal antibody, has been tried in breast cancer, even at low-dose schedules, without significant success, and TKIs have been largely ineffective. Bevacizumab is used in breast cancer for its role in promoting vascular normalization and improving drug delivery in combination with CT and immunotherapy. Additionally, it promotes robust infiltration of immune cells given as a single dose in the neoadjuvant setting (146). To optimize its use, dosing and scheduling require further investigation with imaging/biomarker guidance (Phase Ia). The next phase is a short period of MET induction/epigenetic modifier therapy, e.g., eribulin (Phase Ib). Subsequently, standard neoadjuvant/adjuvant CT is administered, e.g., Adriamycin + Cyclophosphamide (AC), followed by paclitaxel. RT is scheduled to start within six months of surgery, usually after completion of CT cycles (Phase IIa). Targeted therapies such as anti-hormone receptor/anti-human epidermal growth factor receptor 2 (anti-HER2), depending on receptor positivity (Phase IIb). The role of immunotherapy in breast cancer has yet to be strongly defined. However, the tumor is “unmasked” of immune-suppressive elements in the TME, enabling a higher immunotherapy response after Phases I and II. In TNBC, immunotherapy such as nivolumab, pembrolizumab, and atezolizumab (147), or other newer agents, can be tested/retested under the proposed protocol, especially when PD-L1 expression is positive (Phase III). Maintenance therapy is essentially aimed at tackling the sustained proliferative signaling hallmark by targeting hormone receptors/HER2. Depending on clinical indication, poly (ADP-ribose) polymerase (PARP) inhibitors could be part of the schedule (Phase IV) (148).

Final phase: prevention of recurrence strategies, including reducing inflammation in the ECM, keeping the ECM supple, and epigenetic reversion measures. Here, lifestyle diet monitoring (149), senolytics, or metabolic modulators (such as cyclooxygenase-2 (COX-2) inhibitors or metformin) may play a role. In Phase V, immune cell repertoire shrinkage is to be minimized using cell therapies, especially natural killer (NK) cells and *in vitro*-designed therapeutic cancer vaccines, to enhance *in situ* memory cells, including earlier therapy-induced TME-sequestrated tissue-resident memory cells, thereby enhancing long-term immunity (150).

Throughout the above treatment phases, maintaining/enhancing the vasculature, adding phagocytic agents to reduce interstitial pressure, TGF-β blockade to prevent long-term effects of ECM inflammation (12, 151), and immune adjuvants to enhance *in situ* vaccination effects are necessary components. The timing of cell and vaccine therapies, including chimeric antigen receptor (CAR)-NK and CAR-T, needs further evaluation to eliminate residual tumor cells (152, 153).

SBRT at 7–10 Gy per fraction, either as a boost or for oligometastases, is also vascular-normalizing and ECM-preserving, thereby maintaining a supple ECM for immunological cross-talk, given the increasing use of immunotherapy (135, 137). Surgery or SBRT is an integral part of this workflow, with timing determined by clinical indications. Monitoring the therapy protocol requires plasma VEGF and the repertoire of antigen peptides in plasma (immunopeptidomics), which is likely to fine-tune the suggested protocol in the future (72, 145, 154).

## 7 Limitations

The perspectives outlined in the CTS protocol presented in this article may fall short of achieving a definitive cure in all patients. Essentially, the reversion hypothesis suggests that reversing epigenetic changes and associated pathways in transformed, malignant cells may not restore them to their original normal status. EMP finally leaves residual malignant features in one of the forms—DTPs, dormancy, senescent cells, or a “normalized state” (almost normal)—which can always be reactivated to proliferate and induce recurrence, thereby selecting for an evolutionarily more resistant population. Thus, using the CTS strategy to prevent the formation of DTPs, enforcing reversion/differentiation (71), and activating *in situ* vaccine effects (140) become relevant. A combination intermittent senolytic “hit and run” strategy is valid in Phase V, as senescent cells can take weeks to accumulate again after depletion (122).

Therefore, in addition to the steps outlined in Phase V of **Table 3**, further steps beyond the CTS strategy need to be considered. When we presume the cancer is cleared and routine evaluation indicates no evidence of disease, it is imperative to plan the next level of intelligence against a much more battle-hardened, evolutionarily waiting, hidden population (67). At this phase, the complexity of managing heterogeneous, highly adaptable DTPs with intricate feed-forward loops necessitates Systematic Monitoring and Rational Therapeutic Targeting (SMART) approaches, which warrant in-depth study. The available targeting window is dynamic and should coincide with the detection of entry into or exit from the cancer cell cycle, as detected by yet-to-evolve dependable plasma-based molecular methods (72, 154).

## 8 Future research directions

Clinical trials are limited in EMP, despite the vast amount of clinical data (155). Therefore, any effective strategy should include the epigenetic modifiers—”currently the most clinically advanced strategy” (68)—as a potential paradigm shift in targeting cancer cell plasticity and tumor heterogeneity. Monitoring plasma VEGF is promising for the adoption of epigenetic modifiers in clinical practice and for the development of innovative approaches that are found effective in the laboratory (72, 123). Mathematical models should be designed with the pivotal roles of microRNAs and complex signaling pathways in these transitions in mind (156). The emergence of the ability to evaluate the repertoire of antigenic peptides in plasma (immunopeptidomics) offers another frontier for therapeutic monitoring, early diagnosis of molecular recurrence, and a cornerstone for the evolution of robust interventional strategies (154, 157). A comprehensive analysis of these components may reveal potential biomarkers for patient risk stratification and treatment monitoring, as proposed in the present protocol. A highly dynamic, multi-phase, multi-drug protocol of this kind requires, in addition to anatomically based RECIST criteria, high-resolution, real-time molecular feedback to titrate transitions among MTD debulking, MET induction, and evolution-based maintenance. We therefore propose the development and validation of real-time, fluid-biomarker monitoring panels—integrating circulating tumor DNA epigenetic (methylation) profiling, plasma VEGF kinetics, and dynamic plasma immunopeptidomics to effectively implement the CTS framework. In particular, serial immunopeptidomic sampling could track the emergence of new neoantigens generated during Phase III SBRT, allowing the immunotherapy and *in situ* vaccination components to be synchronized to the evolving tumor clonal antigenic landscape (72, 145, 154).

Integrating into the spectrum of these developments, SBRT has the potential to be part of personalized radiation–*in situ* vaccine–immunotherapy, accelerated by nanomaterials, to overcome the hallmark of genomic instability (110, 158). Single-cell sequencing technologies to map the EMT-MET continuum across different cancer types will provide insights into tumor cell population heterogeneity and the influence of varying microenvironments on these transitional states (43, 159).

The lack of routine adoption of EMT pathological characterization, integration into clinical practice, and standardized protocols, despite a vast body of experimental research results and a promising list of drugs, makes its incorporation into the clinical pipeline paramount (155). The advances are in the refinement of trimodal radio-chemo-immunotherapies, awaiting an aggressive, risk-stratified approach, driven by continued technological advances in precision radiation oncology, targeting, and the unraveling of sensitization/resistance molecular mechanisms (94, 128).

An earlier version of the present article is available as a preprint (160).

## 9 Conclusions

Currently, it is a difficult choice between earlier, broad-spectrum, nontargeted approaches with higher toxicity and more recent, increasingly limited-pathway-targeted personalized approaches with excellent response rates in a selected group of patients. Both can increase the risk of resistance for subsequent treatment lines on failure of therapy. This article explores whether the CTS strategy can combine the best of both.

The hypothesis presented here is that tumor heterogeneity and EMP can be therapeutically exploited through strategic combinations, timing, and sequencing, using broad-spectrum therapies complemented with a personalized approach, thereby representing a paradigm shift from viewing EMP as a barrier to recognizing it as a targetable vulnerability. The prerequisite is to normalize vasculature followed by synchronizing cancer cell populations through pharmacological MET induction, normalizing the tumor microenvironment, and preventing evolutionary escape through timed, sequential, non-cross-resistant therapies. The objective is to target primary resistance effectively and prevent the evolution of secondary resistance. The advancing role of RT – SBRT over the decades, from merely cytotoxic cell killing to harnessing synergistic action with immunotherapy, unraveling its potential as a tool for *in situ* vaccination, and the currently expanding role of EMT-MET-Mutational targeting with epigenetic modifiers are integral to the proposed strategy.

The recently reported improvement in overall survival, achieved by combining CT (even when targetable receptors were present) with the initiation of osimertinib (130), exemplifies the combination of broad-spectrum therapy with targeted therapy. The proposed CTS approach integrates the Hallmark concept (91, 92), the immune editing theory (31), Jain’s vascular normalization (133), and Gatenby and Brown’s Evolutionary Therapy approach (40). With accumulated experience using the proposed protocol, it is possible to reduce the intensity and duration of therapy, potentially eliminating toxicities when a risk-stratification approach is adopted.
